# Re-evaluation of the traditional diet-heart hypothesis: analysis of recovered data from Minnesota Coronary Experiment (1968-73)

**DOI:** 10.1136/bmj.q1450

**Published:** 2024-06-28

In this paper by Ramsden and colleagues (*BMJ* 2016;353:i1246, doi:10.1136/i1246, published 12 April 2016), the link to the Broste thesis is outdated. Please see appendix 1 of this correction notice to access the thesis file.

## Web extra

Extra material supplied by authors

Web appendix: Broste thesis

