# Supplementary material for: Re-evaluation of the traditional diet-heart hypothesis: analysis of recovered data from Minnesota Coronary Experiment (1968-73)
Source: BMJ. 2024 Jun 28;385:q1450. doi: 10.1136/bmj.q1450 (PMC11212600; doi:10.1136/bmj.q1450)
Supplement: Supplementary file 1 — Web appendix: Broste thesis [file correction2179.ww.pdf]

STEVEN K.BROSTE M.S.THESIS 1981

MnU-M
81-18

UNIVERSITY OF MINNESOTA
wils,ths
MnU-M 81-18
Broste, Steven Kent.
Lifetable analysis of the Minnesota Coro
3 1951 001 112 354 B
WILSON
ANNEX

THE LIBRARY

REGENTS OF THE UNIVERSITY OF MINNESOTA

LIFETABLE ANALYSIS OF THE MINNESOTA CORONARY SURVEY

A THESIS
SUBMITTED TO THE FACULTY OF THE GRADUATE SCHOOL

BY

Steven Kent Broste

IN PARTIAL FULFILLMENT OF THE REQUIREMENTS
FOR THE DEGREE OF
MASTER OF SCIENCE

March 1981

DEGREE GRANTED
JUN 1981

MnU-M
81-18

This work is dedicated to Cynthia, Nicholas and Christopher, for their patience and understanding. I am grateful to Dr. Glenn Bartsch for his guidance, and to Dr. Ivan Frantz for the use of the Minnesota Coronary Survey data.

TABLE OF CONTENTS

I.	Introduction	1
II.	Epidemiological Studies of CHD	4
III.	The Minnesota Coronary Survey	14
IV.	Baseline Results of the Minnesota Coronary Survey	17
V.	Mortality Results	21
VI.	Life Table Results for Death from Any Cause	40
VII.	Cox Regression Results for Death from Any Cause	50
VIII.	Life Table Results for CHD Death	65
IX.	Cox Regression Results for CHD Death	72
X.	Cox Regression Results for Death from Any Cause, Restricted to Patients Surviving the First Year	81
XI.	Cox Regression Results for CHD Death, Restricted to Patients Surviving the First Year	91
XII.	Summary and Conclusions	102
XIII.	Appendix A	
XIV.	Appendix B	
XV.	Appendix C	
XVI.	Bibliography	

I. INTRODUCTION

Coronary heart disease (CHD) is the leading cause of death in the United States today, accounting for over 600,000 deaths in 1975, according to mortality data from the Vital Statistics of the United States for 1975. Diseases of the heart and blood vessels, which include CHD, account for more than one-half of the total number of deaths recorded in the United States annually. According to the 1975 Annual Report of the Director of the National Heart and Lung Institute, an estimated 3.9 million Americans had CHD, costing the United States economy billions annually in lost productivity and medical costs. Of the heart and blood vessel diseases, CHD is the most common.

It is not surprising then that widespread interest has been focused on the primary prevention of CHD. Primary prevention refers to efforts to prevent the disease from occuring in persons who do not yet have the clinical form of the disease. In order to prevent the disease it is necessary to understand how the disease occurs and to identify possible etiological factors that if treated, offer some hope for prevention.

The coronary arteries are a group of small blood vessels which supply the heart muscle, or myocardium, with blood. The myocardium, like all living muscle tissue, requires an adequate supply of blood to nourish its cells and to eliminate its waste products. If this blood supply is compromised, the myocardium becomes unable to function efficiently, and oftentimes unable to survive. Loss of

efficient pumping action can result in diminished supplies of blood to other vital organs such as the brain and kidneys, and so can result in a host of complications other than the overt heart disease symptoms of heart attack and angina pectoris. The most serious complication of CHD, however, is premature death. Oftentimes, deaths due to CHD are sudden, occuring with few prior symptoms. The disease itself is thought to begin early in life, progressing insidiously through middle age, usually becoming manifest as a heart attack or other illness later in life.

The walls of the coronary arteries, and in fact all human arteries, are made up of three distinct layers. The innermost layer, or intima, is normally smooth and thin. The middle layer, or media, consists of muscle and elastic tissue, while the outer layer, or adventitia, is a fibrous protective covering. The space through which the blood flows is called the lumen, and it is the patency of this space that determines the amount of blood that can be provided to the dependent cells. As long as the inside of the intima remains smooth, elastic, and unobstructed, blood is able to flow relatively unimpeded.

In CHD, however, the lumen of one or more of the coronary arteries becomes reduced in size, causing decreased blood flow to a portion of the myocardium. The primary causes of reduced blood flow in the coronary arteries are arteriosclerosis and atherosclerosis. Arteriosclerosis is the thickening and hardening of the arterial wall, often caused by atherosclerosis, the formation of plaques, or

lipid- rich lesions in the intima. As plaques build up and scar tissue develops, the artery becomes less elastic and more occluded, until blood flow is either halted in one or more of the coronary arteries, or severely restricted. CHD then becomes overt when the reduction or loss of blood supply to the myocardium results in death to muscle cells. The death, or infarction, of a portion of the myocardium causes decreased efficiency or even failure of the entire heart muscle, and often sudden death. In many non-fatal cases, the decreased ability of the heart to pump blood to the rest of the body can result in severe disability and other complications, and usually premature death.

CHD is not strictly a modern disease, although it has been more prevalent in this century than in previous centuries. One reason for the rise in CHD in the industrial era is that life expectancy has increased, sparing people from death due to infectious and other acute diseases, and putting them at increased risk of the more slowly developing, chronic diseases. The disease has been recognized for many years, however, and much of our present knowledge of the epidemiology of CHD is a result of observation and experimentation that took place before the disease had reached its current proportions.

II. EPIDEMIOLOGICAL STUDIES OF CHD

Research into possible causes of CHD has been mainly observational, with the exception of some relatively recent experimental results. The earliest comments regarding atherosclerosis (late 18th century) are gross descriptive accounts of the insides of affected arteries and aortas inspected during autopsy. Moriyama, et. al., (1971) described studies in which researchers examined atherosclerotic plaques in varying stages of development and noted the apparent progression of soft, mushy lesions into hardened plaques. More detailed analyses of the plaques revealed the presence of lipids, including cholesterol. Cholesterol had been identified in the 18th century, and was shown to be present in circulating blood in 1838. When it was found that the atherosclerotic lesions contained high concentrations of cholesterol, a number of questions were formulated. The most basic question was one of cause and effect. It was as yet unknown whether cholesterol caused the lesions or whether the lesions formed independently and somehow collected cholesterol and other lipids from the circulating blood.

The first experimental study on the role of cholesterol and fat rich diets and atherosclerosis was done by Anitschkow (1933) in the early 20th century when diets high in animal fat were fed experimentally to rabbits. It was found that the diets resulted in increased cholesterol levels in the blood and more cholesterol deposited in the walls of the arteries. This animal study did not

nations with typical diets high in animal fat had higher CHD death rates than nations consuming primarily vegetable diets. A few of the studies, such as those described by Katz and Stamler (1953), suggested that countries with high consumption of animal fat had high average levels of serum cholesterol, but generally the international data on serum cholesterol levels were scanty.

In the immediate post World War II period, many studies were undertaken to study further the relationships between diet, serum cholesterol, and CHD. Many of these studies examined international data which had been systematically collected either by the investigators or by internatonal organizations such as the World Health Organization (WHO) and the Food and Agriculture Organization (FAO). Keys (1953) described some of these data, and later was responsible for the Seven Countries Study, which was the most exhaustive international study of diet, serum cholesterol and CHD. Much of this data consisted of food consumption patterns and CHD mortality rates. Nearly all studies showed the now familiar association between dietary intake of fat and cholesterol and CHD. One study, reported by Yerushalmy and Hilleboe (1957), examined the association between national levels of consumption of specific food items such as milk and eggs and CHD mortality. Ironically, much of the diet data was collected by the food industries themselves. Finally, there were some studies, such as the one reported by Kimura (1956), which compared autopsy data on the extent of coronary atherosclerosis between nations, and related the results to dietary

patterns.

While the host of international studies just described presented evidence that would seem to have confirmed the diet/CHD hypothesis many times over, some important evidence was still lacking. Among the important unanswered questions was whether the international diet and mortality data was not merely a reflection of ethnic and cultural factors. To address this issue, studies were made of immigrant populations and corresponding cohorts in the country of origin. Most such studies have shown similarities in the cholesterol levels of both groups, possibly due to the retention of the ethnic diets by the emmigrants, which was suggested by Brown, et. al. (1970) in their study of Irish and Irish American men. A study among Japanese and Japanese Americans, however, showed both increased serum cholesterol (Kagan, et. al., 1974) and increased CHD mortality (Worth, et. al., 1975) in the Japanese Americans.

Similar evidence on the association between changes in diet and changes in serum cholesterol levels has been obtained by examining temporal changes in cholesterol levels and CHD rates within populations. The observation by Malmros (1950) that changes in diet during wartime, necessitated by the war effort, resulted in reduced CHD rates is an example of such evidence. Studies of vegetarians and comparable cohorts of non-vegetarians, such as those reported by Hardinge and Stare (1954) and by Phillips, et. al. (1978) have demonstrated that diets low in or absent of animal fats are associated with decreased CHD mortality.

Most of the evidence presented thus far has involved nations and population groups as the sampling unit. The evidence is impressive from sheer bulk and mainly confirmatory of a link between diet, serum cholesterol and CHD. However, the best evidence to prove the diet hypothesis would be that obtained using individuals as the sampling unit. Prospective studies are the best means to this end, and a number have been done in the past 30 years.

Perhaps the most commonly cited prospective study on CHD, the Framingham Study, provided strong evidence that high levels of serum cholesterol were associated with increased risk of CHD, but did not provide any direct evidence of the role of diet in atherogenesis and CHD. This study followed 5209 members of the adult population of the town of Framingham, Massachusetts beginning in 1948, and continuing for more than 20 years. A great deal of data were collected at various points of time on serum cholesterol levels, and also other possible risk factors. As the population has aged and as both fatal and non-fatal events have occured, the importance of each risk factor to the risk of developing CHD has been assessed. Cholesterol has been identified as one of several important risk factors, along with cigarette smoking, hypertension, and diabetes, as well as other, less important factors. Several similar studies, such as the Albany, Chicago People's Gas Company, Chicago Western Electric Company and Tecumseh studies have, in general, supported these findings. The National Cooperative Pooling Project described by McGee and Gordon (1976), which pooled the results of Framingham and

the four studies just mentioned, demonstrated a strong relationship between serum levels of cholesterol and the subsequent risk of CHD death.

Randomized trials have been undertaken to focus more sharply on the relationship between dietary inake and serum cholesterol. The first of these studies were mostly metabolic ward studies in which experimental and control diets were fed to volunteers under controlled conditions, and cholesterol changes were recorded and related to the content of the diets. These studies demonstrated, for the most part, that it was possible to lower serum cholesterol levels through dietary means.

The studies and observations outlined thus far had established in the minds of most investigators that

a) high dietary intake of cholesterol and fat, particularly saturated fat, was associated with both increased levels of serum cholesterol and increased incidence of CHD

b) increased levels of serum cholesterol were associated with increased risk of CHD in individuals

c) serum cholesterol may be reduced with appropriate dietary intervention.

The picture was as yet incomplete, however. For the previous findings to have practical relevance, it had to be demonstrated that reducing the level of serum cholesterol through dietary change would reduce the incidence of CHD, that is, that CHD was a preventable, delayable, or perhaps even reversible disease. It also had to be

shown that it was possible to modify diets to lower serum cholesterol in a community setting under non-controlled conditions.

A number of intervention trials have been undertaken to try to address these last issues. One such study, reported by Turpeinen (1979), was carried out in two Finnish mental hospitals. The twelve year study, which began in 1959, involved a crossover design in which for the first six years the patients of one hospital received an experimental diet, while the patients of the other hospital received a normal, control diet. After the first six year period the diets in the two hospitals were switched, so that patients receiving the experimental diet in the first period received the control diet in the second period, and vice-versa. The experimental diet was based on no change in total calories or grams of fat, but a greatly modified composition of the fat away from saturated fatty acids toward unsaturated fatty acids. The experimental diet resulted in impressive reductions in serum cholesterol and also in impressive and statistically significant reductions in death due to CHD.

A similar study, the Los Angeles Veterans Administration Domiciliary Study, was also begun in 1959. This trial, as described by Dayton, et. al. (1969), is particularly noteworthy in that it was the first diet study of this kind to be double blind, that is neither the study subjects nor those directly involved in the treatment of the subjects were aware of the diet assignment. The study subjects were middle aged and elderly male veterans living in a veterans home. After various exclusions, 846 participants were

randomized to either the experimental or control diet. As in the Finnish study cited earlier, the experimental and control diets were similar in total calories and in dietary composition, including total fat. The fat composition and dietary cholesterol levels were, however, greatly different, and resulted in a serum cholesterol drop in the experimental group that averaged 12.7% over the course of the trial. Mortality trends favored the experimental group, but due to the small sample sizes the results were not statistic- ally significant, with the exception of fatal atherosclerotic events, a classification made up of a number of diverse events. Because of the advanced age of this population, there is some evidence that the dietary intervention may simply have been "too little, too late".

Realizing the need for a large scale trial to provide a definitive answer to the diet/CHD question, but recognizing the need to assess the feasibility of such a trial first, the National Diet Heart Study (National Diet Heart Study Group, 1968) was undertaken in 1963. This short-term study was also double blind, and had populations drawn from free living middle aged men in five cities, and from the institutionalized population of a hospital for the mentally retarded. A total of 2032 men participated in the study, which employed a number of diets and intervention plans, including a control group. These diets included reductions in saturated fat and dietary cholesterol, and increases in polyunsaturated fat. Various methods of delivery were employed, such as purchase of foods from clinical centers versus purchase on the open market. Again, serum

cholesterol reductions in the diet groups were substantial. The trial was not designed to, and indeed did not, demonstrate an ability to alter the incidence of CHD through dietary intervention. It did, however, develop and suggest methodology for conducting future field trials for this purpose.

For dietary intervention trials, samples drawn from free-living populations pose special problems. Chief among these problems is the lack of control investigators have over compliance to the diet. Adherence even in subjects who have thorough knowledge of the proper diet for reducing serum cholesterol is not likely to be optimal, due to the many outside influences and lack of ready availability of food varieties low in cholesterol and saturated fats. If one is interested in testing the diet hypotheses, then, one is faced with two hard choices. The first alternative is to choose a sufficiently large free-living population so that the small expected changes in serum cholesterol will hopefully result in significant reductions in CHD. The second alternative is to choose a smaller study population in which a larger cholesterol reduction can be achieved, and hopefully a correspondingly large reduction in CHD. CHD death, though it accounts for a larger share of all deaths than any other cause, is still a rather infrequent event, except in some very high risk groups. Thus, using the previously cited studies as models, it would seem desirable to have a fairly large controlled population so that fairly small reductions in CHD mortality could be detected with adequate power and precision. Naturally, the applicability of

results inferred from institutionalized study populations to free-living populations can be severely questioned, but methods aside, it seems reasonable to expect that the basic biological mechanisms at play in institutionalized populations are also applicable to free-living populations. If the diet hypotheses can be proven in institutionalized populations, then there is hope for applying the same theory to other populations, although, intuitively, one would not expect the results to be as impressive.

III. THE MINNESOTA CORONARY SURVEY

It was with these principles in mind that the Minnesota Coronary Survey was begun in 1969. The Minnesota Coronary Survey (MCS) was a double blind trial which utilized the male and female populations of 8 mental hospitals in the state of Minnesota. A recruitment phase was undertaken to randomize all residents of the institutions to either the diet or control groups. Additional patients were randomized to the study during the course of treatment and follow-up as they were admitted to the hospitals. A total of 9570 patients were ultimately randomized to the trial, 4756 in the diet group and 4814 in the control group. One hundred forty-seven of the patients had time on study recorded as zero days, and are excluded from all subsequent analyses. Since the results of this study have not previously been reported in any detail, the results of the MCS will be extensively analyzed here, and will serve as the example for the biometric methods that will be reviewed and developed in what is to follow.

Patients in the study were fed in dining halls, numbered and color coded meal cards being used to assure assignment of the proper tray of food. In addition, the meal cards were punched at each meal to record an index of compliance, since many of the patients had access to other sources of food besides that available in the dining facility.

Baseline variables were collected for each patient during the recruitment phase, including a smoking history, blood pressure

measurements, an electrocardiogram, serum triglyceride and cholesterol determinations, physical examination data and demographic characteristics.

Patients were followed for the possible endpoints listed in Table 1 throughout the course of the trial, and triglyceride and cholesterol values were recorded for each patient still in the study one year after randomization. Because of the nature of the hospitals involved in the MCS, many patients had short periods between admission and release from the hospital, and some released patients were later readmitted to the hospitals. Upon readmission patients were assigned back to their original diet groups. The data on length of stay is summarized in Table 2. Although readmissions were more frequent in the control group than in the diet group, the difference was not statistically significant overall or for either sex. Readmissions were, however, significantly more frequent for males than for females for both study groups.

TABLE 1

Coded Endpoints of the MCS

Non-Fatal Events
================
Acute Myocardial Infarction
Acute CHD Illness
Silent MI by ECG
Acute Stroke

Fatal Events
================
Myocardial Infarction, Autopsied
Myocardial Infarction, Not Autopsied
Cardiac Arrest
Other CHD
Stroke
Other Causes

TABLE 2

Percent of MCS Patients with More than One Admission to the Hospital, by Group Assignment, Age, and by Sex

Age	Diet Group	Control Group
≤ 35	13.6	16.8
35 - 44	10.6	12.0
45 - 54	8.3	7.5
55 - 64	6.0	6.4
> 64	5.7	5.1
TOTAL	9.2	10.1
Males	10.4	11.6
Females	8.1	8.7

IV. BASELINE RESULTS OF THE MINNESOTA CORONARY SURVEY

Some baseline and selected followup characteristics of the two diet groups are displayed in Table 3.

TABLE 3

Some Baseline and Followup Characteristics of the MCS Population by Randomization Group

Variable	Diet Group	Control Group	Diff.
Age at entry (years)	48.2	48.2	0.0
Diastolic Blood Pressure (mm Hg)	76.6	76.7	-0.1
Systolic Blood Pressure (mm Hg)	122.9	123.2	-0.3
Cigarettes Smoked/Day	9.5	9.1	0.4
Percent Cigarette Smokers	43.4	41.5	1.9
Serum Cholesterol (mg/dl)	207.2	207.1	0.1
Serum Triglyceride (mg/dl)	116.6	115.1	1.5
Height (inches)	65.7	65.7	0.0
Weight (pounds)	150.9	150.8	0.1
Body Mass Index (kg./sq.m.)	24.5	24.5	0.0
Percent Male	48.3	48.6	-0.3
Percent with ECG Abnormalities	4.2	4.2	0.0
Percent Diabetic	5.7	5.7	0.0
Percent with Arcus Senilis	19.8	18.9	0.9
Percent with Xanthelasma	2.3	1.9	0.4
Length of Time on Study, Admission 1 (days)	327.0	320.9	6.1
Length of Time on Study, All Admissions (days)	465.9	456.4	9.5
Percent of Meals Eaten	83.8	84.0	-0.2
Percent of Non-breakfast Meals Eaten	85.1	85.2	-0.1

The two groups were comparable on all factors at baseline, and compliance to the diets was also approximately the same. While none of the differences are statistically significant from zero at the

.05 level, the difference in percent cigarette smokers at baseline is almost statistically significant at the .05 level. The age of patients ranged from 20 to 97 years, with mean age 48. Twenty-nine percent of the patients were younger than 35 years of age, and 12% were older than 70. The average age of men in the study was 47.7 years, and for women the average was 48.6 years. For patients who survived one year and remained in the hospital, the average age for men was 51.6 years and for women it was 53.8 years.

Cholesterol and triglyceride were measured on samples collected at six month intervals throughout the study. These samples were measured only for patients who died during the course of the study and for patients who survived the first year and remained in the hospital. Baseline lipid levels were also measured only for this same subgroup. The average of all the 6 month lipid values for each patient will be used here. It is known that nearly all of the lipid changes occured in the first 6 months. Baseline and followup cholesterol and triglyceride are available only on 2489 of the 9423 patients in the study. The average baseline and followup values are given by group and sex in Table 4. The cholesterol drop in the diet group averaged about 15.6%, similar to the reduction achieved in the Finnish study. The reduction in serum triglyceride in the diet group of the MCS averaged approximately 6.2%.

Cholesterol changes were found to be strongly associated with compliance, measured here as the percent of meals eaten in the hospital dining facility. These results are shown in Table 5.

TABLE 4

Cholesterol and Triglyceride Changes, by Randomization Group and Sex

Serum Cholesterol

	Diet Group		Control Group	
	Men(n=674)	Women(n=582)	Men(n=656)	Women(n=577)
Baseline	200.0	217.6	199.0	217.9
Followup	167.6	185.0	192.5	214.7
Change	-32.4	-32.6	-6.5	-3.2

Serum Triglyceride

Baseline	117.7	112.2	114.4	116.5
Followup	107.4	108.5	111.0	121.9
Change	-10.3	-3.7	-3.4	+5.4

TABLE 5

Cholesterol Values at Baseline and Followup for MCS Patients with Both Measurements, by Sex and Percent of Meals Eaten in the Hospital Dining Facility for Patients in the Diet Group

MEN

Compliance Level	n	Baseline	Followup	Change
GE 95%	22	208.5	165.5	-43.0
90-94%	455	200.0	165.6	-34.4
70-89%	146	201.2	172.0	-29.2
LT 70%	51	193.4	173.9	-19.5
	674			

WOMEN

Compliance Level	n	Baseline	Followup	Change
GE 95%	51	236.7	184.0	-52.7
90-94%	375	220.4	183.9	-36.4
70-89%	114	206.9	185.4	-21.5
LT 70%	42	198.9	195.4	-3.5
	582			

V. MORTALITY RESULTS

The MCS data pose a peculiar biometric problem. This is due to the fact that many patients were randomized to the study only to be released from the hospital a short time later. This in itself is not a serious problem with the availability of the various methods for analyzing censored data. However, many of the MCS patients who were released from the hospital were subseqntly readmitted, and placed on their original diet assignment. The problem is then whether to use the information that the patient has survived beyond the first release from the hospital, or whether to ignore all subsequent admissions. Another possibility which seems somewhat attractive is to consider each admission to the hospital as a separate trial of the experiment. This latter possibility had to be eliminated because baseline data was not available for each admission.

Intuitively, it would appear desirable to use all the survival information available. Certainly, summing the total known survival time, including that period of time between admissions when the patient was lost to followup, should be acceptable in the life table analyses, and in regression analyses which include only risk factor levels at entry, and no diet related parameters. This assumes, of course, that the likelihood of being readmitted to the hospital is approximately the same in both diet groups. To illustrate the way in which a bias may be introduced, consider the case in which older patients from the control group and older patients from the diet group are censored in a similar fashion and at a similar rate. Say

now that the censored patients from the diet group experience a much more favorable mortality rate in the subsequent period than the censored patients from the control group. Upon readmission then, it may happen that many of those patients in the control group at highest risk of death will have died while lost to follow-up, and be recorded, not as deaths, but as censored observations. Among patients in the diet group who will be readmitted might be a number whose death has been delayed due to the diet, making it possible for them to be readmitted and to die while on study. Since they would then be recorded as deaths, they might unfavorably affect the mortality comparison between the two groups. It is possible to examine the data for evidence that the rate of readmission differed in the two groups, but no mortality data is available for the patients who were censored and not readmitted. Therefore, use of the readmission data is a somewhat risky venture. Another problem with the use of the readmission data is the watering down of the diet effect. Since the study was double-blind, it is reasonable to assume that if all other factors are equal, patients from both study groups who leave the hospital will have similar diets outside the hospital, and those diets will not necessarily be of the same type as either the control or experimental diets of the study. It is not known how long the diet effect, if it exists, might persist. Therefore, the test of the experimental diet versus the control diet might be weakened by lack of knowledge and control of the diet composition during the lost to follow-up period.

From the discussion above, it would appear to be safer to use only the first admission because of lack of knowledge of the inter-admission period. However, the use of the first admission data only is not without some potential pitfalls. It is very important in a study with heavy censoring such as the MCS that the likelihood of censoring is not associated with the diet assignment. This could bias the mortality comparison in two ways. One possibility is that either the experimental or control diet may improve health to the extent that release from the hospital becomes possible, or impair health to the extent that transfer from the mental hospital to an acute care facility becomes necessary. Either case might result in early censoring for members of one group, who might otherwise have died while on study. The other possibility is that one of the diets may result in some non-fatal event or side effect that could result in either more or less intensive medical care being provided to patients in one of the two groups.

Even in the seemingly "clean" case where only the first admission is considered, then, some understanding of the censoring mechanism is required. Since the MCS patients were randomized over an interval of time, and since the study was officially terminated for all non-censored, surviving patients at one point in time, the potential period of followup in the absence of censoring or death varies from patient to patient. If one now considers censoring to be the event of interest and death to be withdrawal prior to the occurence of the event of interest, then it is possible to study the censoring data

in ways analogous to the ways in which mortality data is analyzed. Since the analysis of the mortality data will receive primary attention, the analysis of the censoring data is considered in Appendix A.

The MCS data represent a larger total sample than any of the studies mentioned previously, although there was heavier censoring and the average length of followup was much shorter. The number of deaths from any cause that were observed in the two groups is shown in Table 6.

TABLE 6

Number of Deaths by Age and Randomization Group

	Diet			Control		
Age	Randomized	Died	%	Randomized	Died	%
LT 35	1367	3	0.2	1337	7	0.5
35-44	728	3	0.4	731	4	0.5
45-54	767	14	1.8	816	16	2.0
55-64	870	35	4.0	896	33	3.7
GE 65	953	190	19.9	958	162	16.9
TOTAL	4685	245	5.2	4738	222	4.7

TABLE 7

Number of CHD Deaths by Age and Randomization Group

	Diet			Control		
Age	Randomized	Died	%	Randomized	Died	%
LT 35	1367	0	0.0	1337	1	0.1
35-44	728	0	0.0	731	1	0.1
45-54	767	4	0.5	816	4	0.5
55-64	870	7	0.8	896	12	1.3
GE 65	953	42	4.4	958	34	3.5
TOTAL	4685	54	1.1	4738	52	1.1

With this first crude look at the mortality data, the results indicate no difference in mortality between the two groups, except for the group 65 years and older. For this subgroup, the diet group has a less favorable mortality experience, the chi-square value for the difference in crude rates being 2.91, not significant at the .05 level.

Table 7 presents the number of CHD deaths by age and randomization group. Once again there is no apparent difference in CHD mortality between the two groups, and the CHD death rate, which overall represents less than 25% of the total deaths, is greater for subjects in the diet group 65 years and older than in the corresponding cohort of the control group. The difference in CHD rates for this older age group is not statistically significant at the .05 level, chi-square being 0.92 in this case. If the diet were to have a favorable effect on mortality, one would expect it to show its strongest benefit in the CHD mortality rates.

The problem of accounting for unequal lengths of follow-up in the two study groups can be addressed by employing one of the life table methods which have been developed to deal with censored survival data.

The types of data collected in clinical trials nearly always include survival information. The term "survival" will be used throughout to denote the event of reaching the end of the period of interest without experiencing an endpoint, whether that endpoint be death or some morbid event. The term "failure" will be used to denote the occurence of the endpoint of interest. The typical prospective clinical trial accumulates subjects for the treatment groups, begins treatment, and follows the subjects for some length of time. This followup period may be the same for all subjects, or may vary from subject to subject. Oftentimes experimental subjects are recruited for a trial over a period of time; thus at any given point in calendar time subjects will have experienced varying periods of treatment and followup. Also, some trials may not be able to follow all subjects until the official termination of the study, particularly in free-living populations or in some hospital studies where subjects may be released from the hospital prior to the end of treatment or followup. Subjects who withdraw from study prior to the full period of followup are termed censored. Censored individuals provide incomplete data on survival. It is known only that they survived that period in which they were under observation, and it is not known whether they survived the entire potential followup

period.

Survival data are frequently presented in the form of life tables, which display the failure experience over the course of the study. Life table methods have been developed which allow the testing for significant differences in mortality between treatment groups, and for displaying mortality data in graphical form.

Two life table methods will be considered. They are the methods described by Kaplan and Meier (1958) and Cox (1972). Before describing these methods, some basic functions used to describe mortality data will be introduced. The cumulative proportion of subjects surviving until at least some time t_i is denoted by $S(t_i)$, where $S(t_0)=1$. The cumulative proportion of subjects failing by t_i is $F(t_i)=1-S(t_i)$.

Another useful quantity to consider is the death density function, denoted by $f(t)$ and defined by

$$f(t) = \lim_{\Delta t \to 0} [Pr(t<T<t+\Delta t)/\Delta t]$$

This function may be described as the probability that an individual dies during the interval $t<x<t+\Delta t$, no matter how small Δt is. The other function which is useful is the hazard rate $\lambda(t)$, defined as

$$\lambda(t) = \lim_{\Delta t \to 0} [Pr(t<T<t+\Delta t | T>t)/\Delta t].$$

The hazard rate is often referred to as the "force of mortality". The hazard function is often used to characterize a survival distribution, a plot of values for the hazard for consecutive intervals often suggesting a parametric distribution for the death

density function.

The first life table technique that will be cosidered here is due to Kaplan and Meier (1958). They introduce the product-limit estimate of S(t) as follows: If the failure times are ordered so that $t_{(1)}<t_{(2)}< \dots <t_{(k)}$, let $n_{(i)}$ be the number of subjects still under study at $t_{(i)}$, including the $\delta_{(i)}$ subjects failing at $t_{(i)}$. Any withdrawals from study at $t_{(i)}$ are considered to have occurred just after $t_{(i)}$. Then

$$\hat{S}(t)= \sum_{j=1}^{k} [(n_{(j)}-\delta_{(j)})/n_{(j)}].$$

If $t_{(i-1)}$ and $t_{(i)}$ are consecutive times to failure, then

$$\hat{S}(t_{(i)})=\hat{S}(t_{(i-1)})[(n_{(i)}-\delta_{(i)})/n_{(i)}].$$

Thus the estimated probability of surviving beyond any observed failure time $t_{(i)}$ may be calculated and put into graphical form. Such graphs can be useful to spot trends in mortality which may suggest more detailed analyses, or a parametric representation for the death density, and hazard rates for intervals may be constructed. Also, the variance of $\hat{S}(t)$ can be used to determine whether differences in survival probabilities between treatments are significantly different from zero. The variance of $\hat{S}(t)$ is approximated by

$$\hat{Var}[\hat{S}(t)]=[\hat{S}(t)]^{2} \sum_{j=1}^{k} 1/[n_{(j)}(n_{(j)}-\delta_{(j)})].$$

The method developed by Kaplan and Meier will be used in the analysis of the MCS data, because use can then be made of the individual times to failure. It is desirable to use all of the survival data which has accrued and to have a method which will give results that are independent of the time intervals chosen. The data will be presented in the form of graphs of the actual life tables,

the life tables themselves being too long for easy interpretation. In cases where the mortality curves for the two groups seem to be different, the data will also be summarized in cumulative annual rates with tests of significance at the end of each one year period.

In the case of randomized trial such as the MCS, balance on possible risk factors is usually achieved by the randomization process, as shown in this case by Table 3. Therefore, one is not compelled to perform regression analyses to adjust for imbalance, but rather one may use regression to investigate whether certain factors are related to mortality. Recently there has been a great deal of interest in the development and refinement of life table methods which make use of additional covariates. Most of the methods that are commonly used introduce covariates into the model in a way that is analogous to multiple linear regression.

The usual procedure is to specify a model for the hazard function, which was defined earlier. The clinical life table was previously suggested as a means to identify the form of the hazard function. In many cases, however, intuition would lead one to try a particular model because of its known properties, its mathematical tractability, or its previous applications.

A model which has frequently been used in cardiovascular epidemiological studies is the logistic model, described and applied by Cornfield, et. al. (1961). The logistic model is often used to model the "risk" of some mortal or morbid event. Risk is usually thought of as the probability of the occurence of some event in a

fixed number of trials or some fixed interval of time. The cumulative distribution function of the death density function, F(t), under the logistic function, defines the risk as

$$F(t;x) = 1/(1+\exp(-xB))$$

where x is a known row vector of independent variables or covariates and B is a column vector of unknown parameters to be estimated from the data. The dichotomous response variable (survive or fail) is known for each subject and it is assumed that all subjects are followed for the entire duration of the study, which is the same for all subjects. If the independent variables x have multivariate normal frequency distributions for both failures and survivors, multiple discriminant analysis using least squares may be used to estimate the coefficients. In the non-normal case an iterative method introduced by Walker and Duncan (1967) can be employed to do the estimation.

The ratio of the estimated regression parameter to its standard error is asymptotically normally distributed, and may be used to assess the statistical significance of each independent variable in predicting response, adjusting for the values of the other independent variables included in the model.

Other models for the survival distribution are the gamma distribution,

$$S(t) = \sum_{\nu=0}^{n-1} \frac{(\lambda t)^{\nu}\exp(-\lambda t)}{\nu\,!}, \quad t \geq 0$$

the Weibull distribution,

$$S(t) = \exp(-\lambda t)^{\gamma}$$

and the exponential distribution,

$$S(t) = \exp(-\lambda t) \ , \quad t \geq 0,$$

where λ is a scale parameter and ν and γ are shape parameters. Each of these survival distributions is the result of a specific representation for the hazard function, that is, an assumption about the force of mortality. The exponential arises from the assumption of a constant hazard $\lambda(t)=\lambda$, the Weibull from the assumption of a hazard $\lambda(t)=\lambda\gamma t^{\gamma-1}$ which may change non-linearly with time, and the gamma from the assumption of a hazard

$$\lambda(t) = \frac{\lambda^n t^{n-1}/(n-1)!}{\sum_{\nu=0}^{n-1} (\lambda t)^{\nu}/\nu!} \ , \quad (\nu \text{ an integer})$$

which may also be non-linear in time. The three models just described do not allow the use of regression like parameters in their current form. However, Feigl and Zelen (1965) considered the scale parameter (i.e. the hazard) of the exponential distribution to be a linear function of a single concomitant variable with an intercept term, such that

$E(t_i) = \lambda^{-1} = a+bx_i$, $i=1,\ldots,n$. In their representation, all subjects were assumed to be followed until death. Given the set of concomitant variables $x_1,\ldots,x_n$ for the n subjects with corresponding death times $t_1,\ldots,t_n$, the estimates $\hat{a}$ and $\hat{b}$ were found using maximum likelihood. The derivation of the normal equations involves setting the derivatives of the log of the likelihood function with respect to the parameters to be estimated

equal to zero. The two resulting equations in two unknowns are solved simultaneously to obtain the parameter estimates. Since the normal equations are not easily solved directly, an iterative technique, usually the Newton-Raphson method, is used.

Zippin and Armitage (1966) extended the results of Feigl and Zelen to the case where not all subjects were followed until death, and also considered multiple concomitant variables. Mantel and Myers (1971) further refined and extended the use of the exponential model.

The basic underlying assumption in using the exponential survival distribution is that the hazard rate is constant. This requirement is a rather restrictive one, especially when a clinical trial to test a treatment is considered. Here, for example, it is likely that patients receiving a beneficial drug might have a better prognosis the longer they are on the drug. In a trial testing a surgical technique, there may be a rather high postoperative risk, but rather good prognosis thereafter.

A model proposed by Cox (1972) has a distinct advantage in that it imposes less severe restrictions on the form of the hazard. His model is analogous to the exponential model extended to allow for censoring and concomitant variables, except that the hazard is modeled as

$$\lambda(t;z) = \exp(zB)\lambda_0(t),$$

where $\lambda(t;z)$ denotes the hazard function conditional on the vector z of covariates. Here $\lambda_0(t)$ is an arbitrary and unknown function of

time, and B is a column vector of regression parameters to be estimated from the data. The great advantage to a model like Cox's is the arbitrariness of $\lambda_0(t)$, the hazard for the standard set of conditions z=0. In his derivation of the likelihood function he requires the analysis to have, as he puts it, "sensible properties whatever the form of the nuisance function $\lambda_0(t)$." Cox suggests that this requirement is perhaps overly cautious, since it is reasonable to assume some smoothness in S(t). He goes on to state that in any case there will result little loss of information about B and that the procedure will likely give one conservative estimates of B. Cox's model can also be used with censored survival times.

Because Cox's method has some important mathematical differences from the methods considered earlier, the derivation will be considered in some detail at this point. Let $t_{(i)}$ denote the i-th ordered failure time of the total of n failure times, and let $m_{(i)}$ denote the number of failures at $t_{(i)}$. $R(t_{(i)})$ will denote the set of subjects at risk of failure at $t_{(i)}$. Then the conditional probability that the failure at $t_{(i)}$ occurs for the subject as observed, given the set $R(t_{(i)})$ of subjects at risk of failure at $t_{(i)}$ is

$$\Pr\{T=t_{(i)} \mid R(t_{(i)})\} = \frac{\lambda_0(t)\exp(z_{(i)}B)}{\sum_{\ell \varepsilon R(t_{(i)})} \lambda_0(t)\exp(z_{(\ell)}B)}$$

$$= \frac{\exp(z_{(i)}B)}{\sum_{\ell \varepsilon R(t_{(i)})} \exp(z_{(\ell)}B)}$$

The above expression gives the contribution of each failure to the likelihood, which is defined as

$$\prod_{deaths} \lambda(t;z) \prod_{all} S(t;z)$$

and thus the likelihood function is

$$\prod_{i=1}^{n} \frac{\exp(z_{(i)}B)}{\sum_{\ell \varepsilon R(t_{(i)})} \exp(z_{(\ell)}B)}$$

and the log likelihood is

$$L(B) = \sum_i z_{(i)}B - \sum_i \log[\sum_\ell \exp\{z_{(\ell)}B\}] .$$

As usual in maximum likelihood estimation, the first and second partial derivatives of the log likelihood function with respect to the parameters B are determined, and the normal equations

$$\frac{\partial L(B)}{\partial B_j} = 0 \quad (j=1,...,k)$$

are solved. The Newton-Raphson method is used to solve the normal equations.

Computationally, one begins with the last ordered failure time $t_{(k)}$ and with some initial estimates of B, accumulating the contributions of that failure time and that of the other subjects at risk at $t_{(k)}$ to the first and second partial derivatives. This is done for each failure time in turn until the derivatives are complete. Thus subjects surviving the maximum followup period provide information for the solution of the normal equations as many times as there are distinct failure times in the sample. The improved estimates of B are then obtained as

$$\hat{B}_i^{(1)} = \hat{B}_i^{(0)} - \frac{[\partial\ L(\hat{B}^{(0)})/\ B_i^{(0)}]}{[\partial^2\ L(\hat{B}^{(0)})/\ B_i^{(0)\,2}]}$$

where the superscript indicates the iteration in which the estimates were obtained, $\hat{B}^{(0)}$ being the initial estimates used in the first iteration. The iterations continue until the difference between successive estimates yields an improvement less than some prespecified level. Since each iteration involves re-evaluating the partial derivatives at the new estimates as described above, the computations can become quite ponderous with large sample sizes and a large number of failures.

The variance-covariance matrix of the estimates $\hat{B}$ is normally obtained as the negative inverse of the expected value of the matrix of second partial derivatives of the log likelihood function with respect to the parameters, evaluated at the final estimates for B. The expected value presents some difficulties here because the censoring times of failures, if they hadn't failed, is unknown. Cox proposes using the values of the second partials as is, with B replaced by $\hat{B}$. Because maximum likelihood estimators are distributed asymptotically as normal, the ratio of the parameter estimates to their standard errors (obtained as the square roots of the elements on the diagonal of the variance- covariance matrix) are approximately normal with mean equal to zero and unit variance. Thus tests of significance regarding the values of $\hat{B}$ can be made. Significance tests about competing parameterizations of the model can also be made by comparing the maximum log likelihoods achieved.

Quite often survival times are grouped in intervals which result in multiple failures (i.e. ties) in some intervals. This can occur

even if the time intervals are recorded in days. When ties occur, some modifications of the technique are required. Specifically, if $m_{(i)}$ subjects fail at time $t_{(i)}$, then the contribution to the likelihood at $t_{(i)}$ involves the conditional probability of the $m_{(i)}$ observed failures occurring, given that $m_{(i)}$ of the $R(t_{(i)})$ subjects at risk at $t_{(i)}$ failed. For example, if $m_{(i)}=2$, index the two deaths as subjects k and l. Then the contribution to the likelihood becomes

$$\frac{\exp\{z_{(k)}B+z_{(l)}B\}}{\sum_{i\neq j}\exp(z_{(i)}B+z_{(j)}B)} \qquad (i,j \in R(t_{(i)}))$$

In the general case with $m_{(i)}$ tied failures at $t_{(i)}$, the contribution can be written as

$$\exp s_{(i)}B \Big/ \sum_{j\in R}\exp s_{(j)}B ,$$

where $s_{(i)}$ denotes the vector of the sum of covariate values for the $m_{(i)}$ individuals failing at $t_{(i)}$ and the summation in the denominator is over all distinct sets of $m_{(i)}$ subjects at risk at $t_{(i)}$. An example will serve to illustrate the difficulties that could be encountered in performing the calculations when ties are involved. Suppose there are 9000 subjects at risk at the time of the first recorded failures. Suppose $m_{(1)}=3$, a situation which is plausible in the MCS with heavy early mortality. Only one addition operation is necessary to compute the numerator of the contribution to the likelihood, but the sum in the denominator must be made over all possible triplets in the risk set of 9000 subjects. This sum has more than 10^{11} terms like the sum in the numerator. The worst possible case is that in which ties occur early in the mortality experience, when the risk

set is large. In the case just described, 9000 terms would have been involved in the summation in the numerator had there been no ties. However, ties occurring late in the experience can also add measurably to the computations required. If two ties occur when the risk set consists of 500 subjects, 61,250 terms comprise the sum in the denominator, compared to the 500 required in the absence of ties. The computations described above are required for each cycle of the iterative process, and so it is clear that an appreciable number of ties can greatly complicate matters. It is possible of course, to save the summations of the covariates for use in iterations after the first, and it is also possible to save the sum of covariates corresponding to the risk set at each distinct failure time. Even with these modifications, a program that would permit calculation of Cox's likelihood in the presence of ties would be complicated and would require an unreasonable amount of computer time.

An approximation of Cox's likelihood proposed by Peto and Breslow in the discussion at the end of Cox's original paper greatly simplifies the computations in the presence of ties. They proposed an approximation to the denominator of the likelihood function of Cox so that the likelihood could be expressed as

$$\prod_{i=1}^{n} \frac{\exp(s_{(i)} B)}{\left(\sum_{\ell \varepsilon R(t_{(i)})} \exp(z_{(\ell)} B)\right)^{m_{(i)}}}$$

with logarithm

$$L(B) = \sum_{i=1}^{n} s_{(i)} B - \sum_{i=1}^{n} m_{(i)} \log\left[\sum_{\ell \varepsilon R} \exp z_{(\ell)} B\right] .$$

This formulation for the likelihood differs from Cox's original formulation only in the denominator. In the original formulation, the denominator is the sum of the hazards of all distinct m-tuples of subjects in the risk set at $t_{(i)}$, with m failures at $t_{(i)}$. In the Peto-Breslow approximation, the denominator is m times the sum of the hazards of all subjects in the risk set. This latter product is approximately the probability of failure in one or more of the risk set, times the number of failures. Since the probability of more than one failure is small if the probability of one failure is small, the approximation seems acceptable on intuition. The derivation of the normal equations for the likelihood as simplified by Peto and Breslow is shown in Appendix B.

The Cox regression model is often referred to as the "proportional hazards" model. This is due to the fact that with the properly chosen parameterization, the estimated regression coefficients can be used to approximate the ratio of the hazard in the control group to the hazard in the experimental group in a simple manner. For example, if an indicator variable takes values 0 and 1 for the experimental and control groups, respectively, then $\lambda(t;1)/\lambda(t;0)$ is the hazard ratio. But

$$\frac{\lambda(t;1)}{\lambda(t;0)} = \frac{\lambda_0(t)\exp(B\cdot 1)}{\lambda_0(t)\exp(B\cdot 0)}$$

$$= \exp(B).$$

This ratio may be estimated using the MLE for B, $\hat{B}$.

VI. LIFE TABLE RESULTS FOR DEATH FROM ANY CAUSE

Figure 1 shows the graph of the Kaplan-Meier life table for the endpoint death from any cause. The abscissa of this graph represents the time, in days, from entry to the trial until death or censoring. The ordinate represents the cumulative proportion of deaths ($\hat{F}(t)$ in the notation introduced earlier), calculated according to the Kaplan and Meier method. The heavy line in Figure 1 represents the mortality experience for the control group, and the lighter line that for the diet group. Each "step" indicates a death time. The vertical distance to the next step is a function of the number of deaths occuring at that time, and also the number of patients at risk at that time. Thus the vertical distance between steps, representing the increase in the cumulative proportion of deaths as a result of one death, can be seen to increase with movement along the abscissa from left to right. This is because there are relatively fewer patients still at risk (ie. alive and uncensored) with succeeding days of follow-up, and so each death has a greater impact on the cumulative rate as time progresses. The horizontal distance between steps represents the time in days between succeeding death times.

Several features are worthy of note in Figure 1. The first feature is the similarity of the mortality curves for the two groups, especially during the first year of follow-up. Following the first year the curves do not separate appreciably, with mortality being generally higher in the diet group, as predicted by the crude

Figure 1

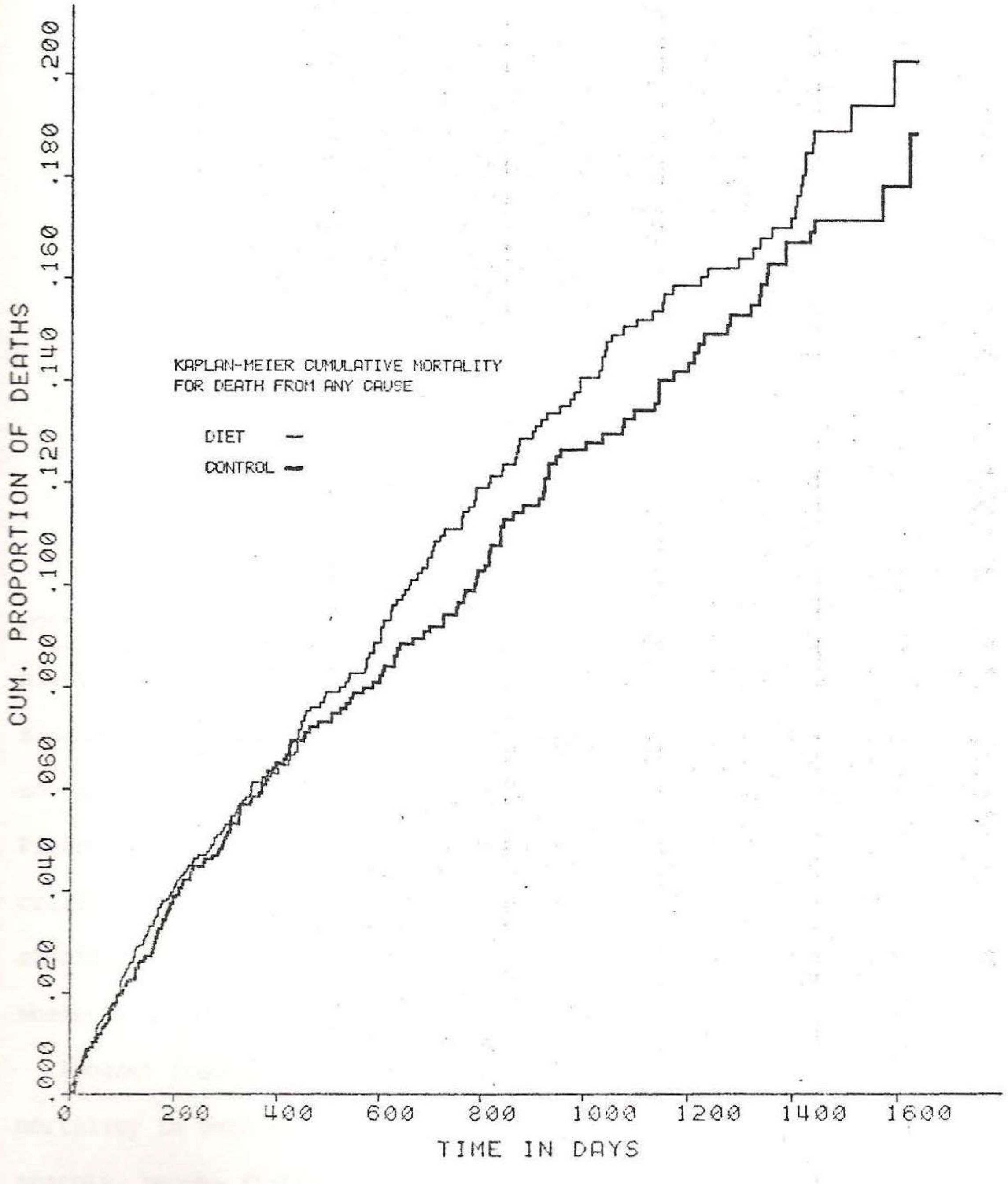

KAPLAN-MEIER CUMULATIVE MORTALITY
FOR DEATH FROM ANY CAUSE
DIET
CONTROL
CUM. PROPORTION OF DEATHS
.000
.020
.040
.060
.080
.100
.120
.140
.160
.180
.200
0
200
400
600
800
1000
1200
1400
1600
TIME IN DAYS

rates presented in Table 6. This observation is further illustrated in the life table summary presented in Table 8.

TABLE 8

Cumulative Mortality as Estimated by Kaplan-Meier For Death from any Cause, by Treatment Group

	Control		Diet		
Interval $t_i - t_{i+1}$ (yrs)	No. at Risk at t_i	$\hat{F}(t_i)$	No. at Risk at t_i	$\hat{F}(t_i)$	Z*
0 - 1	4738	0	4685	0	-
1 - 2	1153	.0591	1165	.0615	-0.32
2 - 3	770	.0940	802	.1109	-1.47
3 - 4	564	.1339	558	.1505	-1.12

* Ratio of difference in cumulative mortality (control-diet) to the standard error of the difference

Although annual intervals were chosen arbitrarily here, and may not be optimal in terms of trying to assess statistically significant differences in mortality, an important difference in a single factor trial such as the MCS should appear and then persist so that it would be apparent in the graph and the summary table. Figure 1 and Table 8 make it clear that the Z-ratio is far from the critical value of 1.96 needed to demonstrate a difference that is significantly different from zero at the .05 level of significance, whatever interval is chosen for the summary table.

Another feature of Figure 1 which is noteworthy is the high mortality in both groups during the first year. Both curves begin steeply, become flatter after one year, and then trace nearly

straight lines thereafter.

The estimated parameter for group assignment for the endpoint death from any cause was obtained using the Cox method. For the same set of patients considered in the life table, $\hat{B} = -0.0960$. Thus the estimated ratio of the hazard in the control group to the hazard in the diet group is exp (-0.0960) = 0.908. This value agrees remarkably well with the ratio of the total number of deaths for the two groups, 222/245 = 0.906. The ratio $\hat{B}/SE(\hat{B}) = -0.97$, not significantly different from zero. This result supports the results of the Kaplan-Meier analysis.

Figure 2 presents results analogous to Figure 1, for males only. For the males, the curves are nearly coincident for the two study groups through about 500 days. Some divergence occurs thereafter, with the diet group experiencing somewhat higher mortality. The males exhibit heaviest mortality in the first year of the study. The Cox regression estimate for the group assignment parameter is -0.0433 for the males, with a Z-ratio of -0.36. The males, representing 4569 of the 9423 patients, contributed 280 of the 467 deaths.

Figure 3 presents the Kaplan-Meier life table for females. As in Figures 1 and 2, there is higher mortality in the diet group, but there is less evidence of the heavy early mortality. The Cox regression estimate for the group assignment parameter is -0.1703 for the females, with a Z-ratio of -1.16. Although the difference between the regression coefficients for males and females is not

Figure 2

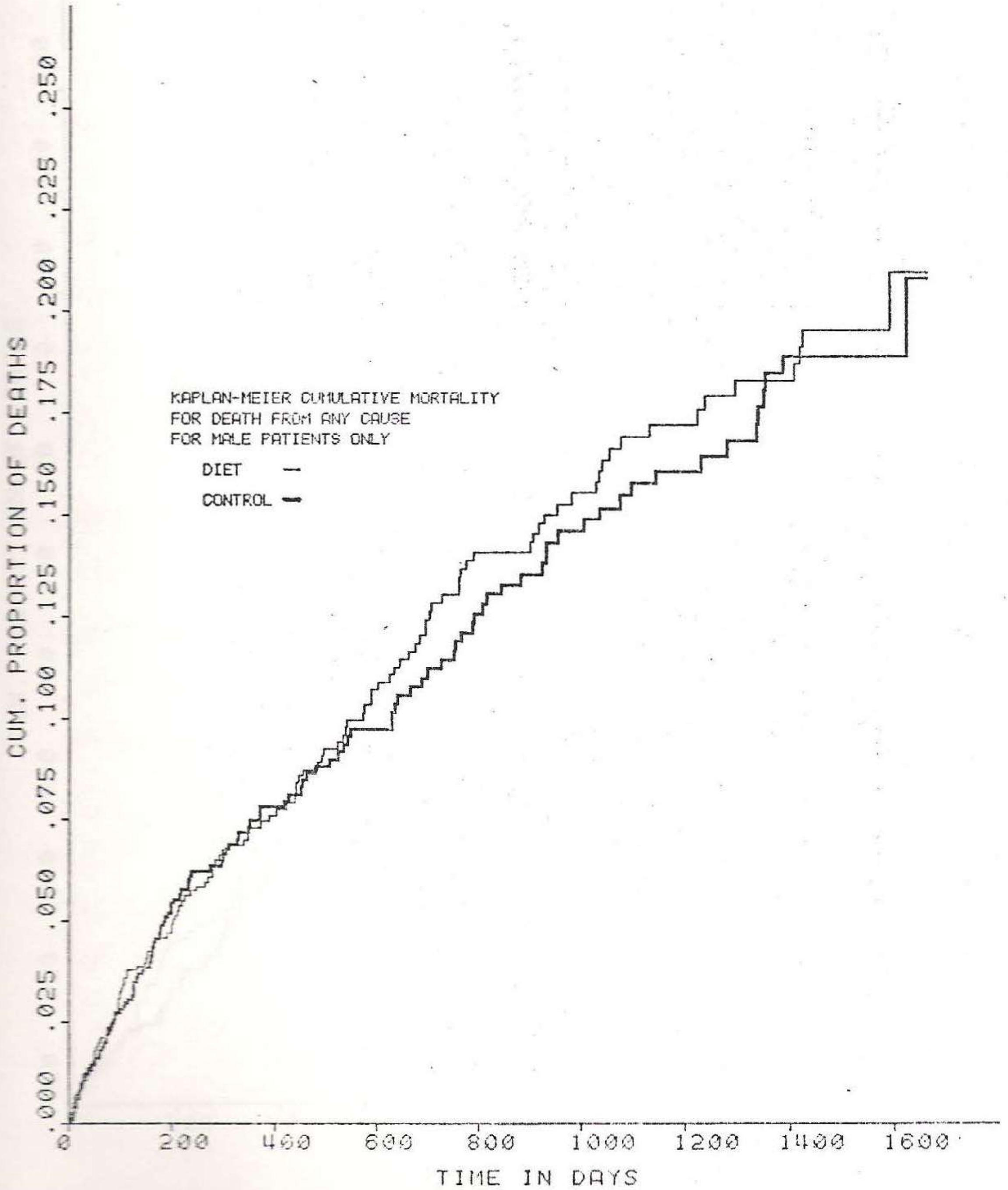
KAPLAN-MEIER CUMULATIVE MORTALITY
FOR DEATH FROM ANY CAUSE
FOR MALE PATIENTS ONLY
DIET
CONTROL
CUM. PROPORTION OF DEATHS
.000
.025
.050
.075
.100
.125
.150
.175
.200
.225
.250
0
200
400
600
800
1000
1200
1400
1600
TIME IN DAYS

Figure 3

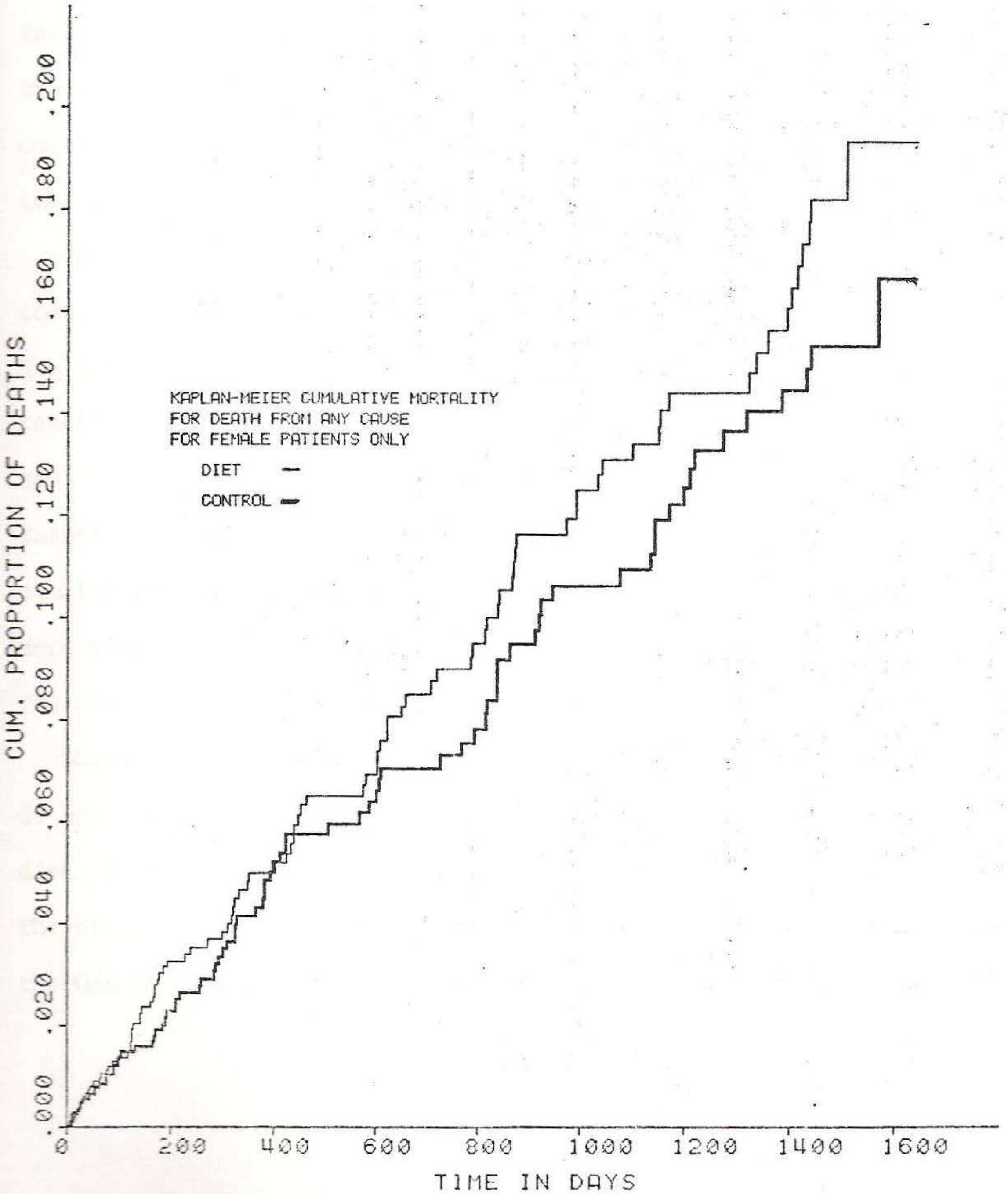

KAPLAN-MEIER CUMULATIVE MORTALITY
FOR DEATH FROM ANY CAUSE
FOR FEMALE PATIENTS ONLY
DIET
CONTROL
CUM. PROPORTION OF DEATHS
.000
.020
.040
.060
.080
.100
.120
.140
.160
.180
.200
0
200
400
600
800
1000
1200
1400
1600
TIME IN DAYS

significant, there is a suggestion that females on the diet fared worse, relative to the controls, than males on the diet.

Table 6 showed that patients under the age of 65 had more deaths in the control than in the diet group. Figure 4 shows the life table for this subgroup, indicating similar mortality experience for the two groups in the first two years, and higher mortality for the control group thereafter. Again, the Cox regression results support this finding, with $\hat{B}$ = 0.1083, and the Z-ratio equal to 0.580.

The excess mortality in the diet group seems to have been confined primarily to patients 65 years or older, again as seen from Table 6. Figure 5 displays the life table for this subgroup, and the results are further summarized in Table 9. The graph shows rather similar mortality for the two groups up to about 400 days, and rather remarkable divergence thereafter. The difference in cumulative mortality for the two groups is 2.08 standard errors from zero after two years, in spite of dwindling sample size. The Cox regression parameter in this case is -0.1924, with Z-ratio = -1.80.

Obviously, the findings of the MCS with regard to total mortality do not exhibit evidence of a beneficial effect of the experimental diet. It has been shown that mortality is related to sex and to age. The effect of these and other factors will be further explored using the Cox regression approach.

Figure 4

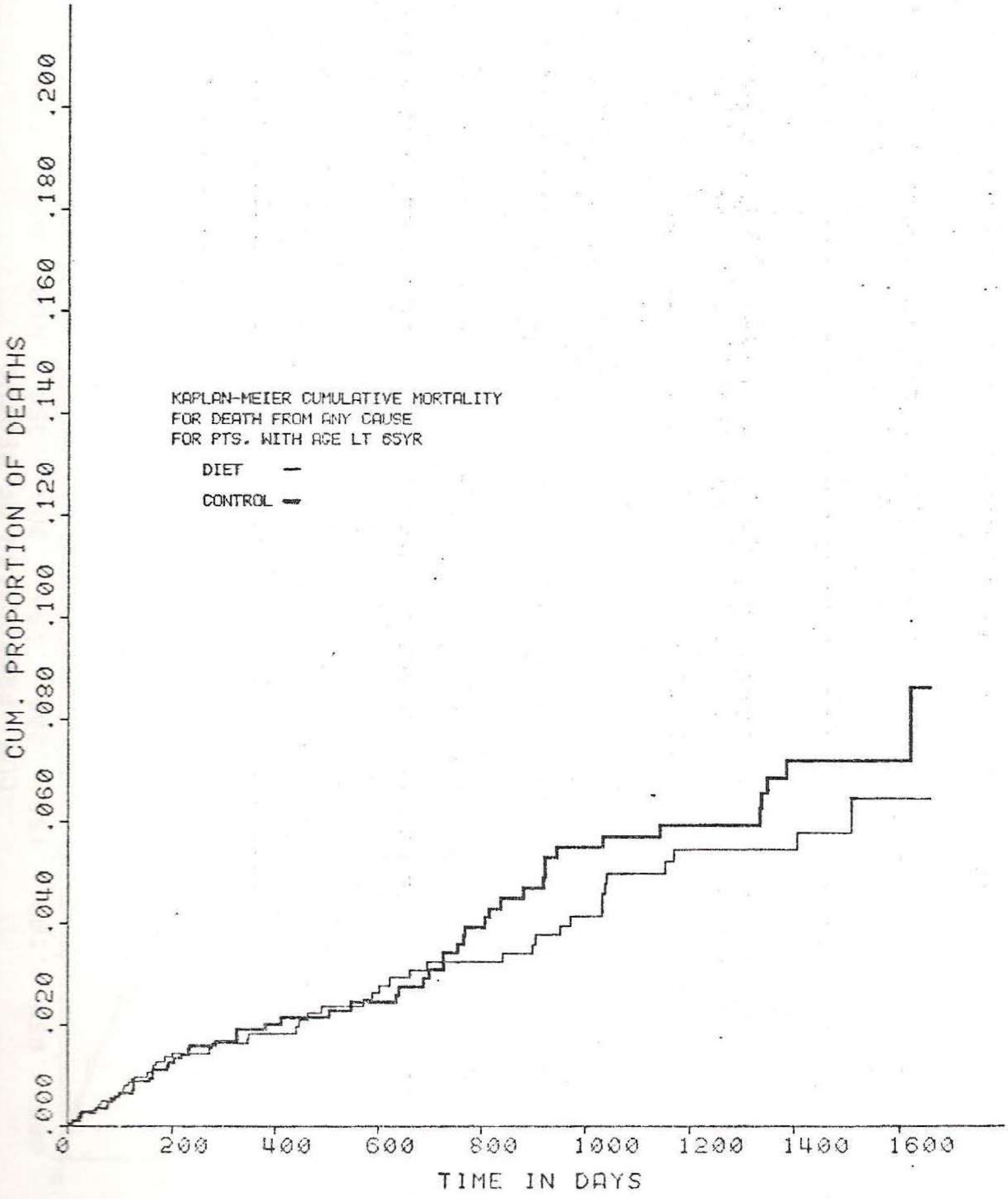
KAPLAN-MEIER CUMULATIVE MORTALITY
FOR DEATH FROM ANY CAUSE
FOR PTS. WITH AGE LT 65YR
DIET
CONTROL
CUM. PROPORTION OF DEATHS
.000
.020
.040
.060
.080
.100
.120
.140
.160
.180
.200
0
200
400
600
800
1000
1200
1400
1600
TIME IN DAYS

Figure 5

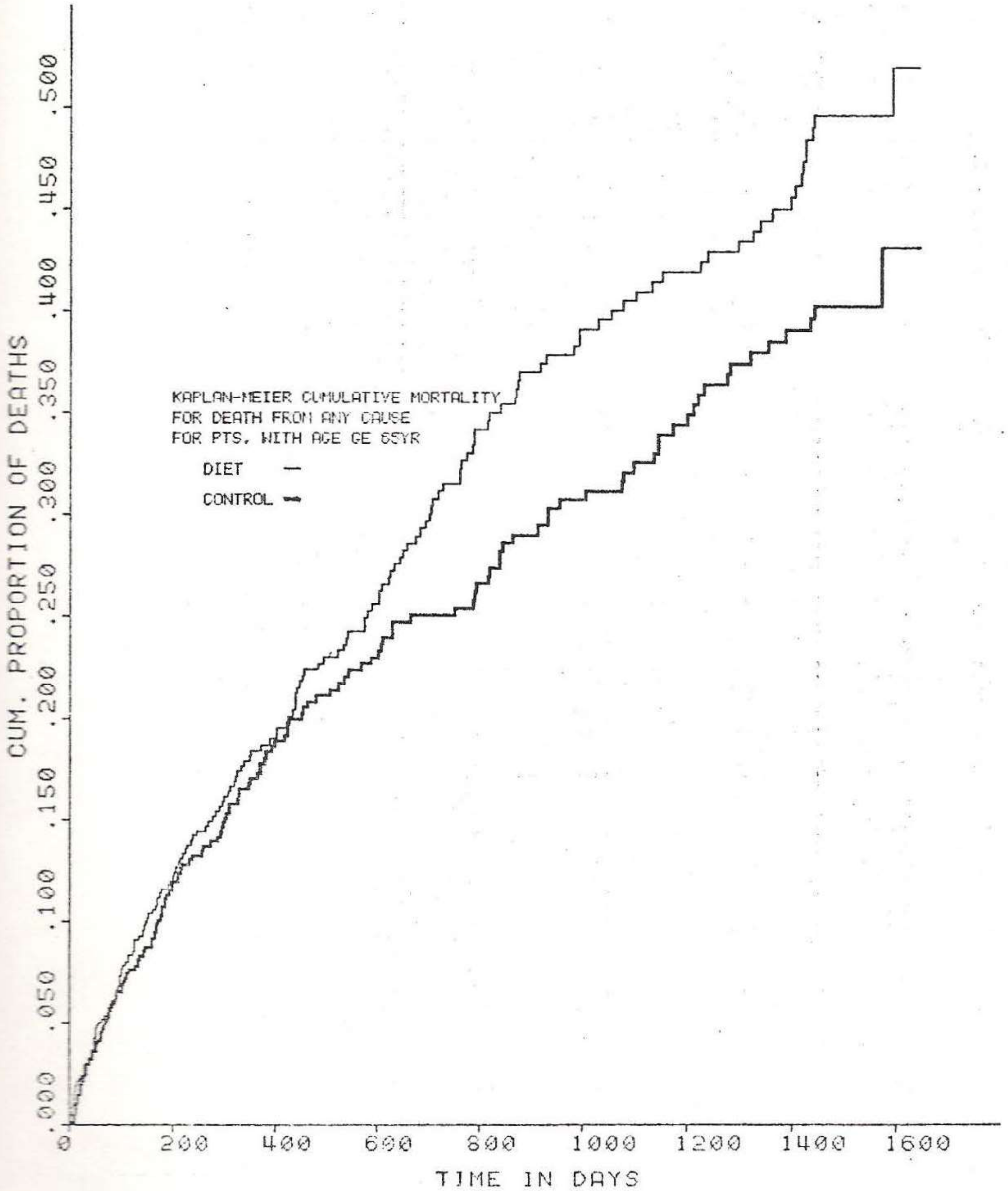
CUM. PROPORTION OF DEATHS
.000
.050
.100
.150
.200
.250
.300
.350
.400
.450
.500
KAPLAN-MEIER CUMULATIVE MORTALITY
FOR DEATH FROM ANY CAUSE
FOR PTS. WITH AGE GE 65YR
DIET
CONTROL
0
200
400
600
800
1000
1200
1400
1600
TIME IN DAYS

VII. COX REGRESSION RESULTS FOR DEATH FROM ANY CAUSE

As indicated previously, the Cox regression method may be used to study the effects that various factors or covariates have on mortality. A number of the traditional risk factors for CHD and total mortality will be included in these analyses, including age, diastolic blood pressure (DBP), cigarettes per day, body mass index (BMI), sex, and presence or absence of diabetes. BMI is a measure of relative body weight, expressed in kilograms of body weight per meter of height squared. In addition, it is of interest to study a number of treatment variables. Randomization group was used as the sole covariate in the previous section, and will be used here as well. Another treatment variable is the percent of meals which were not eaten in the hospital dining facility. Some patients were able to obtain meals outside of the hospital, and thus were not totally compliant to their assigned diet. Baseline cholesterol and triglyceride are also of interest, but no data on either were available for patients who left the hospital before the first year anniversary of their randomization. The impact of cholesterol and triglyceride and the changes in these variables will be studied in a later section which will be restricted to patients still in the hospital after one year.

Preliminary analyses, which will not be shown here, indicated that there might be interactions between group assignment and some of the covariates. For this reason, all models will include

interaction terms for group assignment and age, BMI, sex, and percent of meals missed. With these interaction terms included in the models, care is needed in interpreting the results. For example, if a model containing group assignment, age, and the age x group assignment interaction were fitted, with 0 indicating the diet group and 1 indicating the control group, then the estimated ratio of hazards for the two treatment groups, assuming age was equal in the two groups, would be

$$\frac{\hat{\lambda}(t|z_1=1)}{\hat{\lambda}(t|z_1=0)} = \exp(\hat{B}_1+\hat{B}_3 z_2)$$

where $\hat{B}_1$ is the coefficient estimate coresponding to the group assignment parameter, z_2 is the variable representing age, and $\hat{B}_3$ is the coefficient estimate corresponding to the interaction. It can be seen that the ratio of hazards is now allowed to change with age. If the estimate for $\hat{B}_3$ were close to zero, one would conclude that the effect of age on mortality is approximately the same for both treatment groups, although age might well be an important factor related to mortality. If the estimate for $\hat{B}_3$ were large, one would conclude that age affected mortality differently for the two groups. If the coefficient were positive, the conclusion would be that increasing age increased mortality more in the control than in the diet group, and a negative coefficient would suggest that increasing age increased mortality more in the diet than in the control group.

With one or more group interaction included in the model, the interpretation of the group assignment parameter becomes difficult.

One might consider evaluating the hazard ratio at various values of the covariates included in the interactions, but the group assignment parameter by itself has no useful interpretation. The interpretation of the other parameter estimates included with group assignment in the interactions is also more difficult than in the case with no interactions. In the above example where age was used as an interaction with group, $\hat{B}_2$, the coefficient estimate for age would represent the effect of age for the diet group. $\hat{B}_2+\hat{B}_3$ would represent the effect of age for the control group.

The life table and regression analyses of the preceding section indicated that the diet group experienced less favorable mortality than the control group. This result was unexpected, and so the rather complicated model described above was chosen to give some insight into what might have caused this excess of mortality in the diet group.

Table 10 shows the result of fitting the model with the interaction terms for all patients who had complete data. The coefficient estimates will be discussed in detail for each parameter for this first and most basic model. The group assignment parameter may be ignored because it is uninterpretable due to the interactions.

The coefficient estimate for the age x group interaction has a Z-ratio of -2.00, indicating that the effect of age is significantly less in the control group than in the diet group. The coefficient estimate for age has a Z-ratio of 14.30, larger than for any of the

TABLE 10

Cox Regression Results for the Endpoint
Death from Any Cause

Variables	Coefficient Estimate	Z-ratio
Group Assignment 0=Rx,1=Control	2.1435	2.56
Age	0.0783	14.30
Diastolic Blood Pressure	-0.0034	-0.79
Cigarettes/Day	-0.0365	-3.73
Body Mass Index	-0.0357	-2.31
Diabetes 0=No,1=Yes	-0.0010	-0.00
Sex (0=F,1=M)	0.6122	4.32
Percent of Meals Missed	0.0031	0.38
Age x Group	-0.0153	-2.00
BMI x Group	-0.0525	-2.17
Sex x Group	0.1600	0.78
% Meals Missed x Group	-0.0139	-1.10

Maximum log likelihood achieved = -2883.202

Total N = 9021, number of fatal events = 407

other coefficient estimates. In the diet group, it is estimated that aging 10 years would increase the hazard by a factor of $[\exp(0.0783)]^{10}$, or 2.19. In the control group, aging 10 years would increase the estimated hazard by $[\exp(0.0783-0.0153)]^{10}$, or a factor of 1.88.

Elevated diastolic blood pressure has been associated with increased mortality in many studies. In the MCS, the coefficient for DBP is negative, indicating that there is an inverse relationship between DBP and mortality. The coefficient estimate is less than one standard error from zero, however.

In nearly all prospective studies of more or less general populations, cigarette smoking has been associated with increased mortality. In the MCS, there is an inverse relationship between smoking and mortality, and the coefficient is highly significant, as shown in Table 10. It can be argued that the MCS is not a typical population, however, and it may be true that those patients who were most severely ill were unable to obtain or smoke cigarettes while in the hospital. The heavy early mortality seen in the life tables is suggestive of many severely ill or very aged patients dying early in the study. More detailed discussion of this finding will be deferred until the summary, as it is unlikely that smoking rates were different between the two study groups, or that diet interacted with smoking.

The coefficient estimate for the BMI x group interaction is statistically significant, indicating that BMI had a differential

effect in the two groups. The coefficient estimate for the main effect of BMI is also significant. Both the main effect and interaction have negative signs, indicating that mortality was highest among the relatively light patients, and that this negative effect was more pronounced in the control group. This finding is also consistent with the possibility of high mortality among patients who were rather ill and debilitated at entry. The significance of the interaction coefficient would suggest that the diet had somehow improved prognosis for the patients with low BMI. Again, more detailed discussion of the BMI results will be deferred until the summary.

Presence or absence of diabetes was not significantly associated with mortality. The sex x group interaction is positive, but not significantly different from zero. Sex, however, had a strong association with mortality with a Z-ratio of 4.32. The positive coefficient estimate indicates that mortality was highest among males, with males in the diet group estimated to have a hazard 1.8 times that of the females in the diet group. For the control group, the ratio of hazards is approximately 2.2.

The final result in Table 10 is for the percent of meals missed. The interaction of group and percent of meals missed is negative, but not significant. For the diet group, missing meals was associated with increased mortality, but the coefficient is very near zero. For the control group, the combining of the coefficients suggests that compliance with the control diet was negatively

associated with mortality. Perhaps this compliance index measures different things in the two groups. In the control group, the ability to eat outside the hospital may be a measure of relatively good health. The control diet was to be a more or less "normal" diet, and would presumably not be expected to improve prognosis. The experimental diet, on the other hand, may have improved prognosis so that compliance with it would have resulted in more favorable mortality experience for those patients who were unable to leave the hospital than for those who could. Table 5 showed that compliance to the experimental diet resulted in impressive reductions in cholesterol.

Since sex was shown to be an important factor in the life tables and the previous regression, it was decided to do separate regressions for males and females. Table 11 presents the results for all causes of mortality for males, and Table 12 presents the results for females. The results of Tables 11 and 12 are somewhat different.

For males, the age x group interaction is statistically significant, and for females it is still negative, but not significant. The coefficient estimates for the main effect of age are also somewhat different, the estimate being larger for the males. In both sexes, the effect of aging was estimated to be more severe in the diet group.

For males, the interactions of BMI and group, and percent of meals missed and group are both negative, the Z-ratio exceeding 1 in both cases, but falling short of statistical significance. The main

TABLE 11

Cox Regression Results for the Endpoint
Death from Any Cause for Male Patients

Variables	Coefficient Estimate	Z-ratio
Group Assignment 0=Rx,1=Control	2.6876	2.40
Age	0.0896	11.99
Diastolic Blood Pressure	-0.0046	-0.81
Cigarettes/Day	-0.0255	-2.51
Body Mass Index	-0.0589	-2.49
Diabetes 0=No,1=Yes	0.0231	0.11
Percent of Meals Missed	-0.0033	-0.29
Age x Group	-0.0241	-2.40
BMI x Group	-0.0401	-1.17
% Meals Missed x Group	-0.0267	-1.42

Maximum log likelihood achieved = -1541.802

Total N = 4338, number of fatal events = 242

TABLE 12

Cox Regression Results for the Endpoint
Death from Any Cause for Female Patients

Variables	Coefficient Estimate	Z-ratio
Group Assignment 0=Rx,1=Control	1.3526	1.05
Age	0.0647	7.78
Diastolic Blood Pressure	-0.0006	-0.09
Cigarettes/Day	-0.1048	-2.71
Body Mass Index	-0.0205	-0.99
Diabetes 0=No,1=Yes	-0.0719	-0.29
Percent of Meals Missed	0.0159	1.21
Age x Group	-0.0038	-0.30
BMI x Group	-0.0573	-1.66
% Meals Missed x Group	0.0031	0.17

Maximum log likelihood achieved = -1058.169

Total N = 4683, number of fatal events = 165

effects of both factors are also negative, with BMI showing a Z-ratio of -2.49, and percent of meals missed not significant. Taking the estimates at face value and ignoring the statistical significance, there is a suggestion that for the males, as for all patients combined, compliance and low BMI were associated with relatively poorer prognosis for the control group patients than for the diet patients.

For females, the coefficient estimate for the interaction of BMI and group, and percent of meals missed and group are not significant, although the BMI interaction has a Z-ratio of -1.66. For females, the estimate for the main effect of BMI is negative, as in the previous regressions, but not significant. The coefficient estimate for percent of meals missed is positive, but only 1.21 standard errors from zero, suggesting improved prognosis for female patients in both groups who complied to their assigned diets.

The best fitting of two or more competitive models may be determined by comparison of the maximum log likelihoods achieved. The ratio of the likelihood of the expanded model to the likelihood of the reduced model is a likelihood ratio statistic, Λ . If the estimated likelihood ratio is 1, that implies that the additional parameters, or the stratification, have not improved the fit. But $-2 \ln \Lambda$ is distributed as chi-square with degrees of freedom equal to the difference in the number of parameters in the two models. For the data presented in Tables 10, 11, and 12, $\Lambda = \exp(-283.2)$ and $-2 \ln \Lambda = 566.5$, with 8 degrees of freedom. Obviously, stratification

by sex has greatly improved the fit over the single model presented in Table 10.

Since both the life tables and the previous regressions showed that the effect of age on mortality was more severe in the diet group, and since age was the variable with the largest Z-ratio in the previous regressions, the data were stratified by age. Sixty-five years was selected as the cutoff for this stratification because it left a moderate number of fatal events in the younger stratum, and because the total population over 65 was large enough to result in reasonable precision in that stratum. Tables 13 and 14 display the Cox regression results for patients less than 65 and 65 or older, respectively.

For both strata, the age x group interactions were negative, although not significantly so for the older stratum. The coefficient estimates for the main effect of age were positive and significant for both strata, the coefficient being larger for the younger stratum. Aging is again estimated to have a more severe impact on the diet patients.

For both strata, cigarettes per day has a negative and statistically significant coefficient, as in the previous analyses. For both the patients younger than 65 and those 65 or older, DBP has a low negative coefficient estimate.

For the younger stratum, the interaction of sex and group is essentially zero, indicating that the excess mortality in males was of approximately the same magnitude in both study groups. For the

TABLE 13

Cox Regression Results for the Endpoint
Death from Any Cause for Patients Younger Than 65 Years

Variables	Coefficient Estimate	Z-ratio
Group Assignment 0=Rx,1=Control	4.0469	2.59
Age	0.0758	4.71
Diastolic Blood Pressure	-0.0065	-0.72
Cigarettes/Day	-0.0416	-2.84
Body Mass Index	-0.0083	-0.28
Diabetes 0=No,1=Yes	-0.1121	-0.30
Sex (0=F,1=M)	0.3473	1.16
Percent of Meals Missed	-0.0042	-0.28
Age x Group	-0.0401	-1.97
BMI x Group	-0.0740	-1.62
Sex x Group	-0.0077	-0.02
% Meals Missed x Group	-0.0170	-0.81

Maximum log likelihood achieved = -711.708

Total N = 7245, number of fatal events = 99

TABLE 14

Cox Regression Results for the Endpoint
Death from Any Cause for Patients 65 Years or Older

Variables	Coefficient Estimate	Z-ratio
Group Assignment 0=Rx,1=Control	1.6875	1.19
Age	0.0599	5.75
Diastolic Blood Pressure	-0.0029	-0.59
Cigarettes/Day	-0.0260	-1.99
Body Mass Index	-0.0422	-2.32
Diabetes 0=No,1=Yes	-0.0071	-0.04
Sex (0=F,1=M)	0.6641	4.09
Percent of Meals Missed	0.0049	0.55
Age x Group	-0.0118	-0.76
BMI x Group	-0.0469	-1.64
Sex x Group	0.2870	1.21
% Meals Missed x Group	-0.0122	-0.80

Maximum log likelihood achieved = -1935.398

Total N = 1776, number of fatal events = 308

older stratum, the sex x group interaction is larger and over one standard error from zero. The positive sign of this coefficient suggests that the excess mortality in males is somewhat greater in the control group. Males in both study groups fared worse than the females for both strata.

It is rather difficult to sort out the meaning of the coefficients when both factors in the interaction are binary variables, as in the case of sex and group. For example, females (coded 0) result in terms equal to zero for both sex and sex + sex x group, regardless of study group. It would be incorrect to assume from this that the effect of being female is the same in both groups. Rather, one should compute the relative hazard of being male in one group to being female in the same group. These relative hazards may then be compared for the diet and contol groups, as was done above.

For both of the age strata, the coefficient estimate for the BMI x group interaction is negative and 1.6 standard errors from zero. The coefficient estimate for the main effect of BMI is nearly zero for the younger patients, but much larger and statistically significant for the older patients. This supports the hypothesis that the large negative effect of BMI in Table 10 may have been due to debilitation among the older patients. Although not significant, the interaction coefficients again suggest that BMI was more of a negative factor among the control patients.

The interaction terms for the percent of meals missed are

negative for both strata, with Z-ratios of -0.8 for both. Neither coefficient estimate for the main effect of the percent of meals missed is more than one standard error from zero, although the sign is negative for the younger stratum and positive for the older stratum. In both strata, compliance, or whatever is being measured by this variable, is estimated to be less beneficial for control patients, and perhaps beneficial only for patients in the older stratum who were on the experimental diet. The low significance levels of all the coefficient estimates involving compliance in these two strata give one little faith in these results, however.

As in the stratification by sex, the improvement in fit of the model stratified by age can be assessed via the likelihood ratio statistic. In this case, $-2 \ln \Lambda = 472.192$, with 8 degrees of freedom, again indicating an improved fit with the stratification.

VIII. RESULTS FOR CHD DEATH

Since the experimental diet used in the MCS was designed to reduce serum cholesterol, and hopefully to retard or reverse the process of atherosclerosis, one would have hoped that the diet would have shown a beneficial effect on the incidence of CHD death. The crude CHD mortality rates in Table 7 indicated no beneficial effect on this endpoint, although the excess CHD mortality in the diet group consisted of only 2 events. Figure 6 and Table 15 present the Kaplan-Meier life table for this endpoint, confirming the lack of any important difference in CHD mortality between the two groups. The Cox regression estimate for the group assignment parameter is -0.0127, with a Z-ratio of -0.07, further supporting the lack of a CHD mortality difference between the two groups.

Figures 7 and 8 present the life tables for CHD death for males and females respectively. When the small number of CHD deaths is further divided into strata, the curves are difficult to interpret. No clear trend is apparent, as the lines cross one another in both graphs. The males seem to have experienced somewhat heavier CHD mortality in the early part of the study than in the later part. The Cox regression estimates for the group parameter are 0.0478 (Z=0.20) for males and -0.1229 (Z=-0.37) for females.

Figures 9 and 10 display the life tables for the CHD death endpoint for patients younger than 65 and 65 or older, respectively. There were so few CHD deaths in the younger stratum that one must conclude that the treatment groups are indistinguishable. In the older stratum, the patients

Figure 6

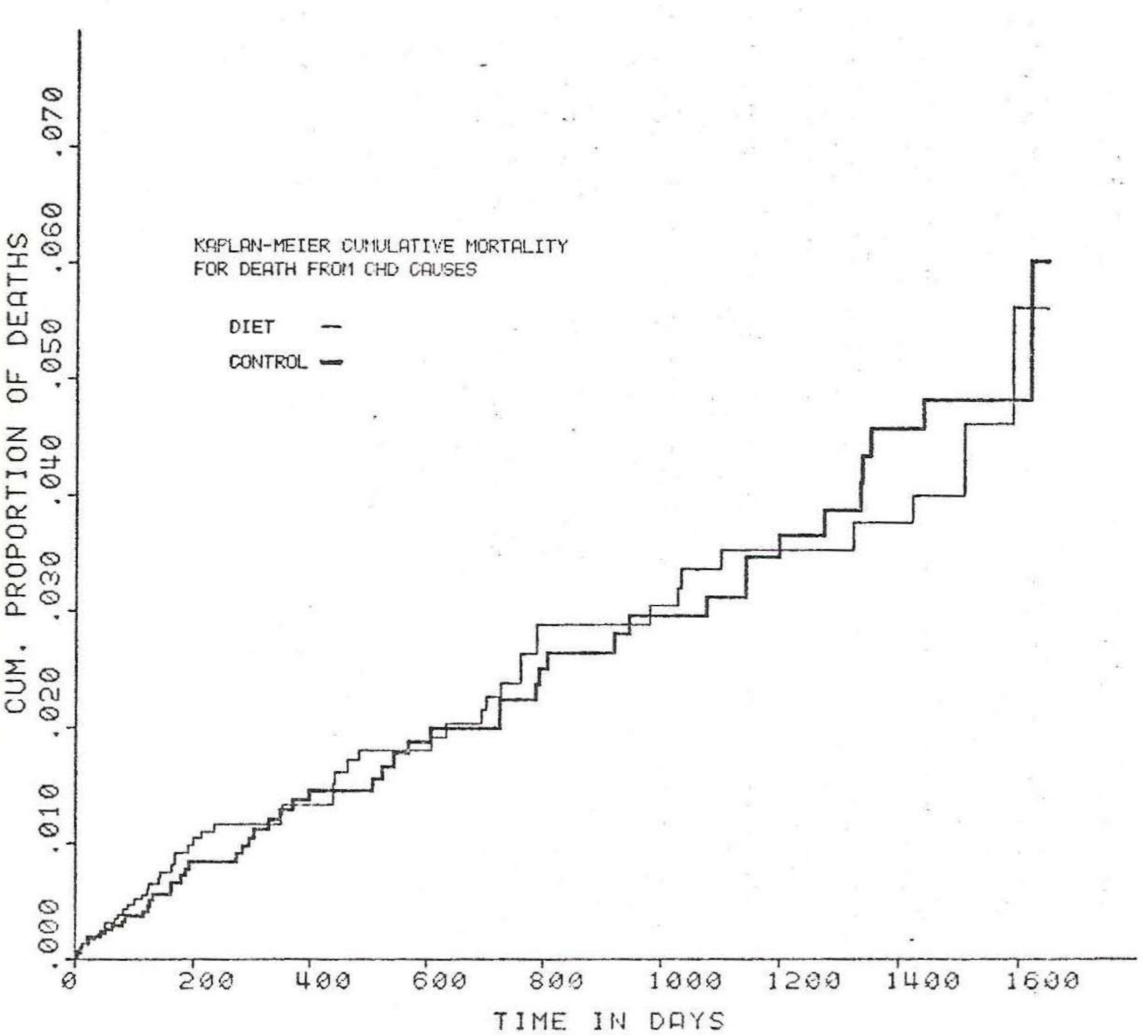
KAPLAN-MEIER CUMULATIVE MORTALITY
FOR DEATH FROM CHD CAUSES
DIET
CONTROL
CUM. PROPORTION OF DEATHS
.000
.010
.020
.030
.040
.050
.060
.070
0
200
400
600
800
1000
1200
1400
1600
TIME IN DAYS

Figure 7

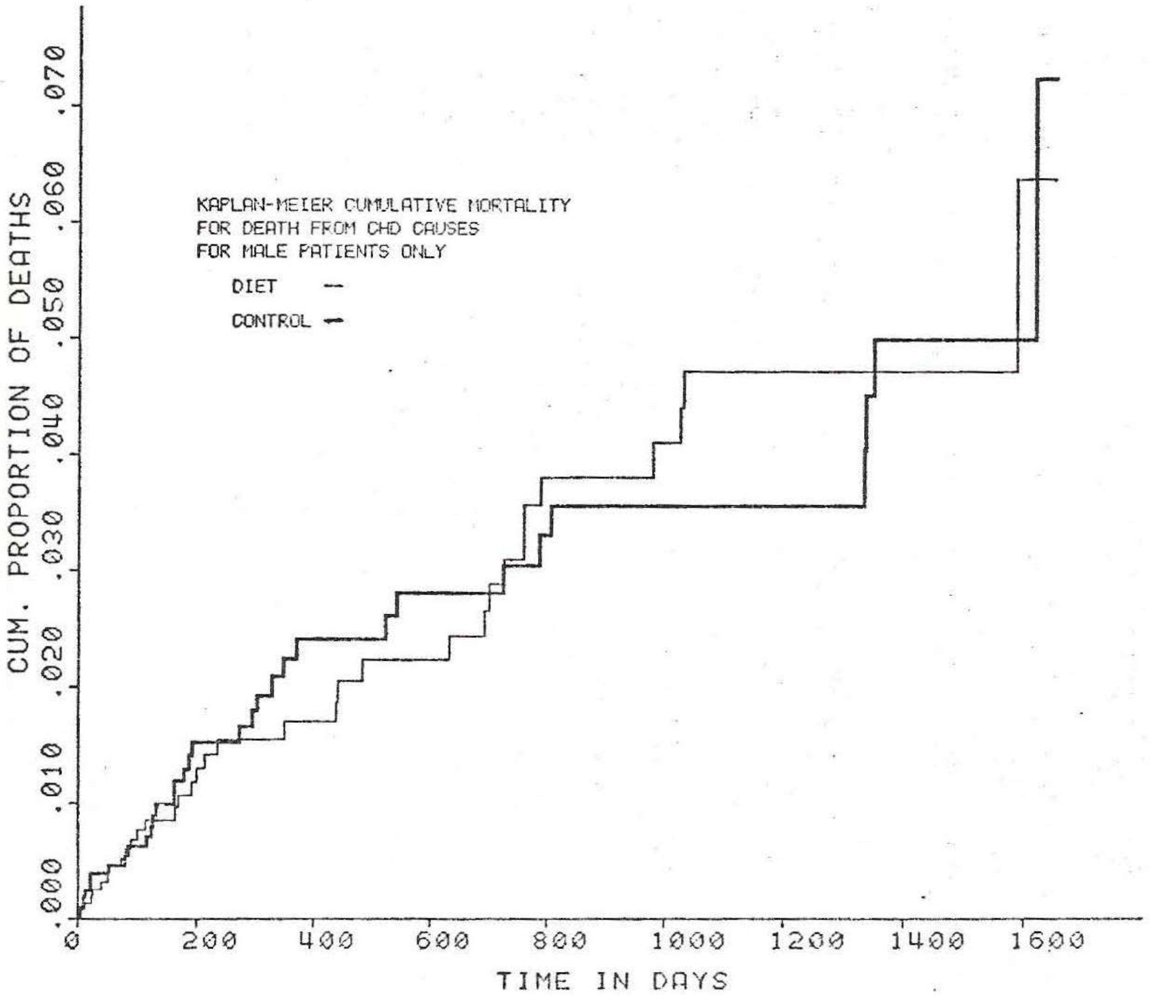

KAPLAN-MEIER CUMULATIVE MORTALITY
FOR DEATH FROM CHD CAUSES
FOR MALE PATIENTS ONLY
DIET
CONTROL
CUM. PROPORTION OF DEATHS
.000
.010
.020
.030
.040
.050
.060
.070
0
200
400
600
800
1000
1200
1400
1600
TIME IN DAYS

Figure 8

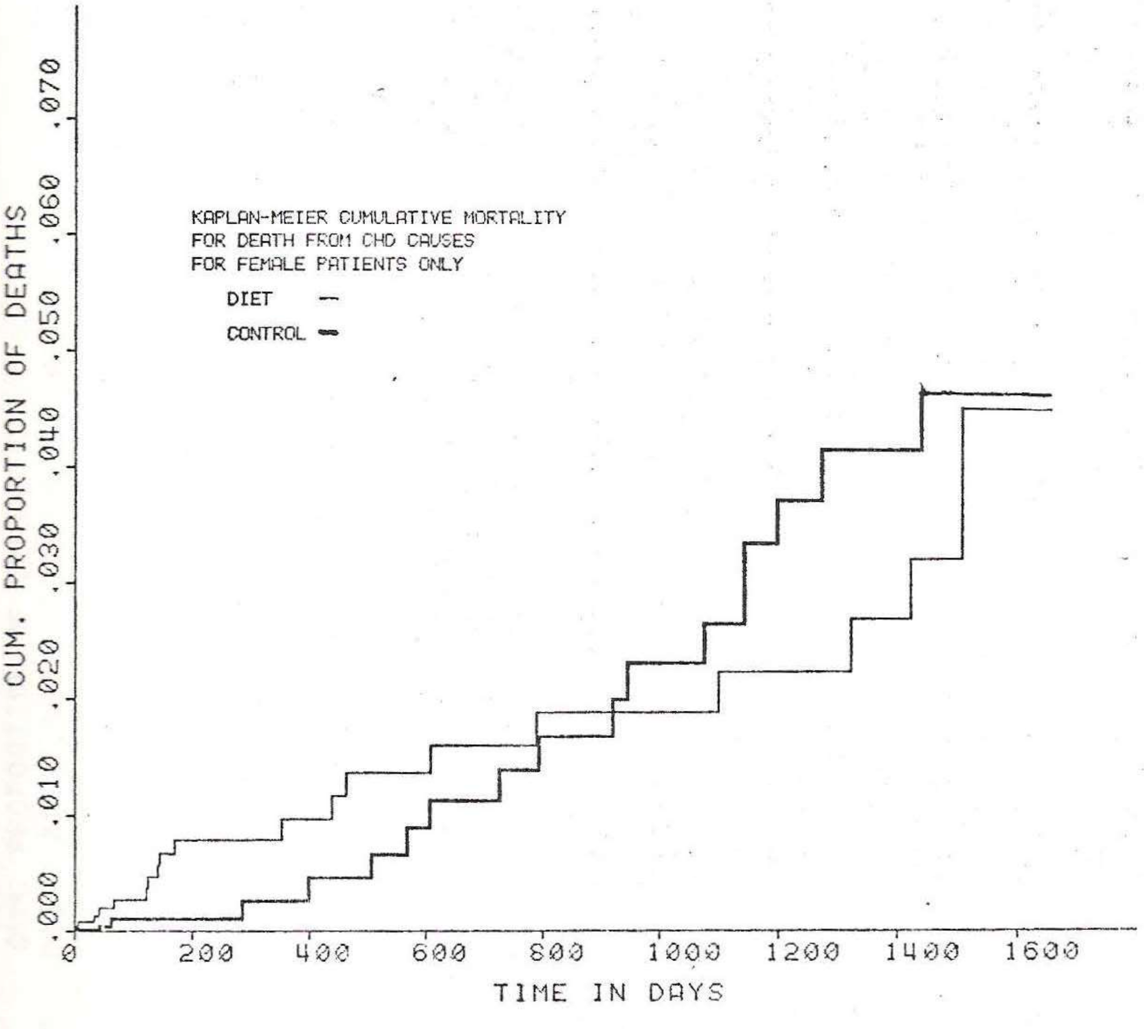
KAPLAN-MEIER CUMULATIVE MORTALITY
FOR DEATH FROM CHD CAUSES
FOR FEMALE PATIENTS ONLY
DIET
CONTROL
CUM. PROPORTION OF DEATHS
.000
.010
.020
.030
.040
.050
.060
.070
0
200
400
600
800
1000
1200
1400
1600
TIME IN DAYS

Figure 9

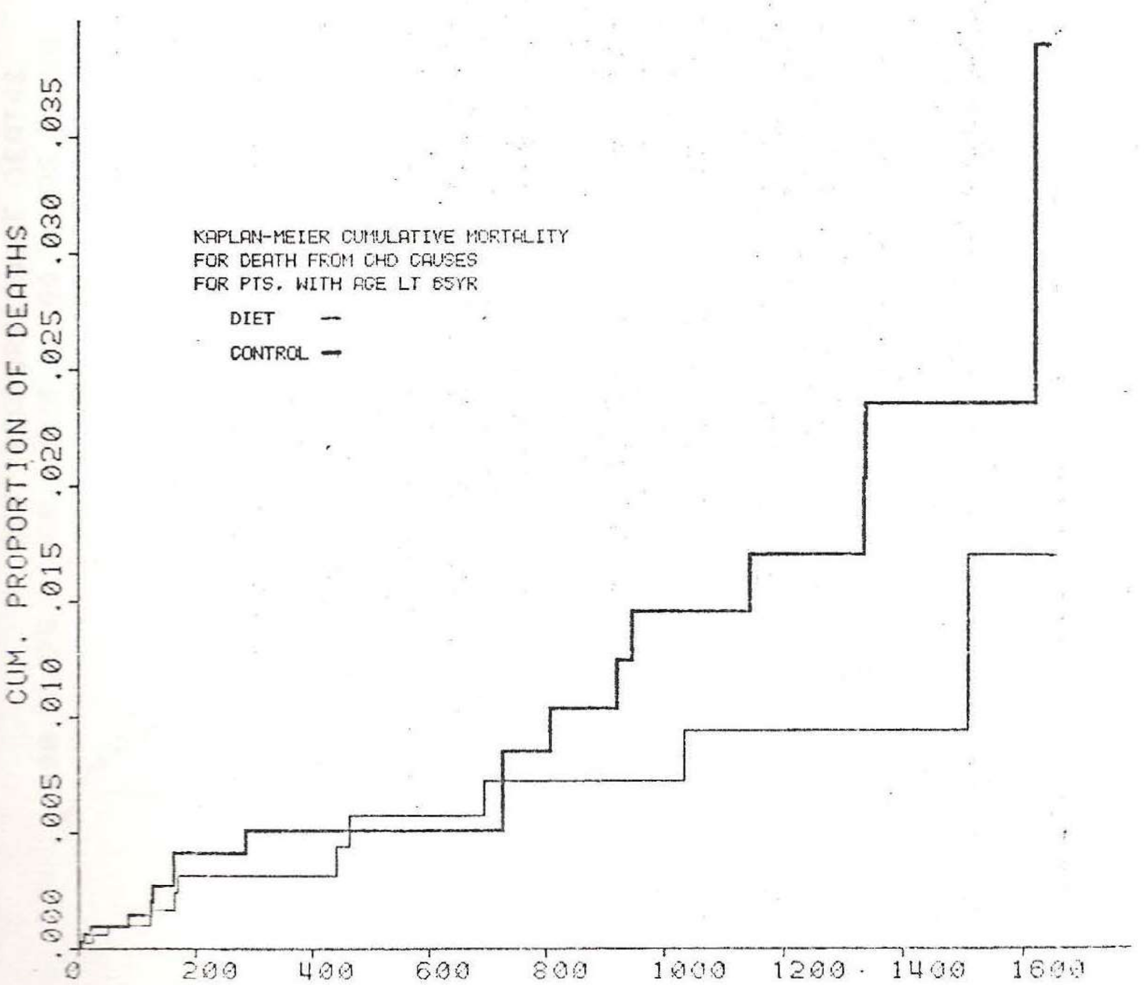
KAPLAN-MEIER CUMULATIVE MORTALITY
FOR DEATH FROM CHD CAUSES
FOR PTS. WITH AGE LT 65YR
DIET
CONTROL
CUM. PROPORTION OF DEATHS
.000
.005
.010
.015
.020
.025
.030
.035
0
200
400
600
800
1000
1200
1400
1600
TIME IN DAYS

Figure 10

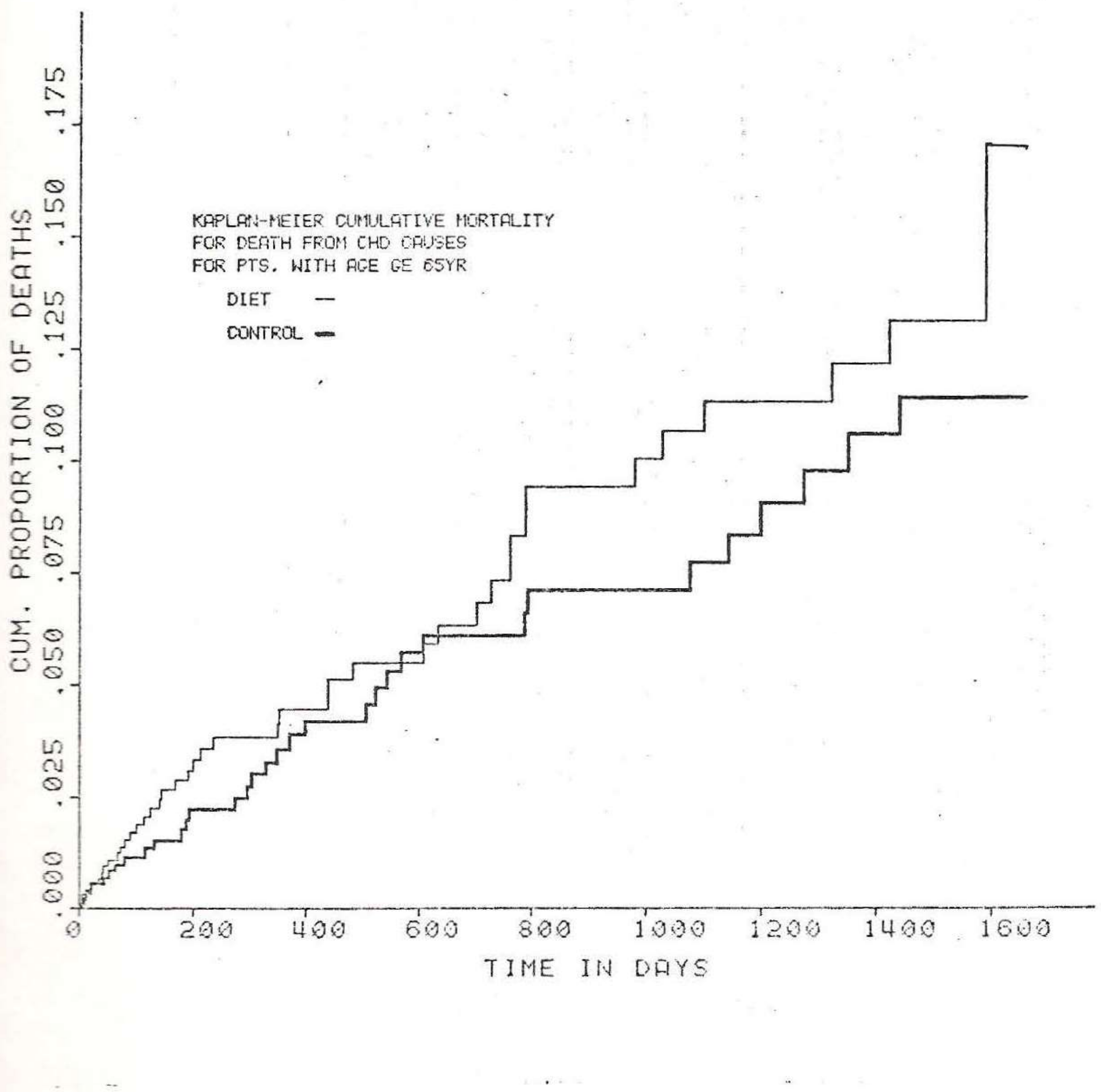

KAPLAN-MEIER CUMULATIVE MORTALITY
FOR DEATH FROM CHD CAUSES
FOR PTS. WITH AGE GE 65YR
DIET
CONTROL
CUM. PROPORTION OF DEATHS
.000
.025
.050
.075
.100
.125
.150
.175
0
200
400
600
800
1000
1200
1400
1600
TIME IN DAYS

in the diet group experienced consistently higher CHD mortality, though the difference was not statistically significant at any of the annual intervals. Furthermore, most of the difference resulted from the experience in the first year, in which 25 diet group patients and 19 control group patients died of CHD.

TABLE 15

Cumulative Mortality as Estimated by Kaplan-Meier For CHD Death, by Treatment Group

	Control		Diet		
Interval t_i-t_{i+1}(yrs)	No. at Risk at t_i	$\hat{F}(t_i)$	No. at Risk at t_i	$\hat{F}(t_i)$	Z*
0 - 1	4738	0	4685	0	-
1 - 2	1153	.0128	1165	.0134	-0.16
2 - 3	770	.0224	802	.0239	-0.25
3 - 4	564	.0312	558	.0336	-0.31

* Ratio of difference in cumulative mortality (control-diet) to the standard error of the difference

IX. COX REGRESSION RESULTS FOR CHD DEATH

Table 16 presents the Cox regression results for the complete model with interaction terms for the CHD death endpoint. The results for CHD death are rather similar to those presented earlier for death from all causes. The coefficient estimate for the age x group interaction is negative, but not significant, and the coefficient estimate for the main effect of age is significant and positive, as before. Diastolic blood pressure is not significantly associated with CHD death, although the sign of the coefficient is positive. Cigarette smoking is negatively associated with CHD mortality, although the coefficient estimate is only 1.16 standard errors from zero.

The BMI x group interaction, while not quite significant at the .05 level, suggests that the relationship between CHD mortality and BMI is in the negative direction and much stronger for the control group than for the diet group. This latter observation is consistent with that for all causes of death. The coefficient estimate for the main effect of BMI, which was consistently and significantly negative for all causes of mortality, has a positive but not significant coefficient estimate for CHD death. The coefficient estimate for BMI, which was consistently and significantly negative for all causes of mortality, has a positive but not significant coefficient estimate for CHD death for the diet group.

Diabetes was not associated with increased mortality from all causes, but is somewhat related (Z=0.82) to CHD mortality. The

TABLE 16

Cox Regression Results for the Endpoint
CHD Death

Variables	Coefficient Estimate	Z-ratio
Group Assignment 0=Rx,1=Control	3.9403	2.14
Age	0.0801	6.21
Diastolic Blood Pressure	0.0025	0.27
Cigarettes/Day	-0.0230	-1.16
Body Mass Index	0.0119	0.36
Diabetes 0=No,1=Yes	0.2495	0.82
Sex (0=F,1=M)	0.7851	2.44
Percent of Meals Missed	-0.0652	-1.53
Age x Group	-0.0253	-1.47
BMI x Group	-0.0926	-1.84
Sex x Group	0.2408	0.53
% Meals Missed x Group	-0.0229	-0.37

Maximum log likelihood achieved = -605.348

Total N = 9021, number of fatal events = 88

association is in the direction of increased CHD mortality for diabetics.

The coefficient estimate for the interaction of sex and group is positive, but not significant. The coefficient estimate for the main effect of sex is also positive, with a Z-ratio of 2.44.

Compliance with the assigned diet was associated with higher mortality in both groups, and although the interaction is not significant, slightly more so in the control group.

While the small number of CHD deaths limits the precision of the estimates, it is interesting to note that the CHD death results are somewhat similar to those for death from all causes, even though the CHD deaths represent only about 25% of the total number of deaths.

Tables 17 and 18 show the Cox regression results for CHD death for males and females respectively. The results for males are very similar to the results for both sexes combined, shown in Table 16. All of the coefficient estimates have the same sign in both tables. The age interaction for both sexes, while not significant, indicates that the effect of aging was more severe in the patients in the diet group. The puzzling positive association between BMI and CHD mortality in the diet group, and the negative effect in the control group, as evidenced by the significant interaction coefficient, are present for males. For females, the coefficient estimates for neither the interaction nor the main effect of BMI are significant.

In males, the coefficient estimate for the percent of meals missed x group interaction is negative, and 1.31 standard errors

TABLE 17

Cox Regression Results for the Endpoint
CHD Death for Male Patients

Variables	Coefficient Estimate	Z-ratio
Group Assignment 0=Rx,1=Control	4.9336	2.14
Age	0.0723	4.47
Diastolic Blood Pressure	0.0055	0.49
Cigarettes/Day	-0.0167	-0.76
Body Mass Index	0.0388	0.83
Diabetes 0=No,1=Yes	0.1298	0.32
Percent of Meals Missed	-0.1609	-1.83
Age x Group	-0.0183	-0.85
BMI x Group	-0.1366	-2.04
% Meals Missed x Group	-0.2224	-1.31

Maximum log likelihood achieved = -353.575

Total N = 4338, number of fatal events = 57

TABLE 18

Cox Regression Results for the Endpoint
CHD Death for Female Patients

Variables	Coefficient Estimate	Z-ratio
Group Assignment 0=Rx,1=Control	3.7446	1.24
Age	0.0938	4.16
Diastolic Blood Pressure	0.0009	0.06
Cigarettes/Day	-0.0370	-0.67
Body Mass Index	-0.0198	-0.41
Diabetes 0=No,1=Yes	0.3749	0.80
Percent of Meals Missed	0.0158	0.38
Age x Group	-0.0424	-1.40
BMI x Group	-0.0319	-0.42
% Meals Missed x Group	-0.0112	-0.21

Maximum log likelihood achieved = -188.058

Total N = 4683, number of fatal events = 31

from zero. The main effect of this factor is also negative, with a Z-ratio of -1.83. This suggests that compliance was detrimental for both groups, but particularly for the control group. For females, compliance seemed to be of little importance for the CHD death endpoint.

Tables 19 and 20 present the Cox regression results for patients younger than 65 years and 65 or older, respectively. These tables confirm the previous findings with regard to age, sex, DBP and cigarette smoking. For younger patients, the coefficient estimate for the BMI x group interaction is negative and significantly different from zero. The estimate for the main effect of BMI is positive, but not significant, suggesting that BMI was a negative risk factor for younger patients in the control group, but was positively related to CHD mortality for patients in the diet group. The coefficient estimates for the BMI x group interaction and the BMI main effect are both negative for the older patients, but not significant.

For the younger stratum there is a suggestion that diabetes might be a risk factor, patients with diabetes having an estimated hazard more than twice that of non-diabetics. The coefficient is only 1.62 standard errors from zero, however.

For the younger patients, the percent of meals missed was not associated in any powerful way with CHD mortality. For patients 65 years or older, the coefficient estimate for the interaction term for the percent of meals missed is negative and 1.2 standard errors

TABLE 19

Cox Regression Results for the Endpoint
CHD Death for Patients Younger Than 65 Years

Variables	Coefficient Estimate	Z-ratio
Group Assignment 0=Rx,1=Control	12.3528	2.46
Age	0.1591	2.31
Diastolic Blood Pressure	-0.0054	-0.28
Cigarettes/Day	-0.0396	-1.27
Body Mass Index	0.0971	1.44
Diabetes 0=No,1=Yes	0.8442	1.62
Sex (0=F,1=M)	1.0409	1.26
Percent of Meals Missed	-0.0013	-0.03
Age x Group	-0.0991	-1.33
BMI x Group	-0.2248	-2.32
Sex x Group	-0.4615	-0.47
% Meals Missed x Group	-0.0323	-0.58

Maximum log likelihood achieved = -147.616

Total N = 7245, number of fatal events = 23

TABLE 20

Cox Regression Results for the Endpoint
CHD Death for Patients 65 Years or Older

Variables	Coefficient Estimate	Z-ratio
Group Assignment 0=Rx,1=Control	1.4091	0.46
Age	0.0434	1.92
Diastolic Blood Pressure	0.0052	0.50
Cigarettes/Day	-0.0096	-0.37
Body Mass Index	-0.0053	-0.14
Diabetes 0=No,1=Yes	-0.0112	-0.03
Sex (0=F,1=M)	0.7295	2.06
Percent of Meals Missed	-0.1368	-1.55
Age x Group	-0.0038	-0.11
BMI x Group	-0.0617	-1.04
Sex x Group	0.5724	1.09
% Meals Missed x Group	-0.2266	-1.20

Maximum log likelihood achieved = -398.989

Total N = 1776, number of fatal events = 65

from zero. The coefficient estimate for the main effect is also negative, and over 1.5 standard errors from zero. This again suggests that compliance was somewhat more beneficial for diet patients, although the estimates would suggest that compliance with either diet was associated with higher CHD mortality.

X. COX REGRESSION RESULTS FOR DEATH FROM ALL CAUSES, RESTRICTED TO PATIENTS SURVIVING THE FIRST YEAR

As indicated earlier, the MCS diet was designed to reduce serum cholesterol in the diet group, thereby hopefully reducing death from CHD and also death from all causes. Lipid analyses were performed on blood collected at entry only for those patients who died or who were alive and still in the hospital at the end of the first year. This was done in an effort to reduce the expenses of the study, since it was felt that patients leaving the hospital in the first year would not provide much information on the efficacy of the diet. Previous analyses suggested that the diet did not improve prognosis for the entire cohort. It is of primary importance to study whether baseline lipid levels were associated with mortality, and also whether changes in lipids were associated with mortality. This latter question is essentially the contribution of the MCS to the testing of the diet/CHD hypothesis.

Many studies, some of which were described earlier, have demonstrated that elevated serum cholesterol levels were associated with increased mortality, particularly CHD mortality. There is some speculation that triglyceride levels might also be associated with mortality, but previous studies have not shed much light on this issue. Therefore, the Cox regression models were expanded to include baseline cholesterol and triglyceride, and also the changes in these factors. One might have considered expressing the lipid and lipid change parameters as main effects and interactions with treatment

group, but there were several reasons for not doing so. The primary reason was that similar changes in cholesterol should result in comparable changes in risk of death, regardless of how the change in cholesterol was achieved. Secondly, the addition of four more interactions would complicate the interpretation of the model, now restricted to a greatly reduced sample. The analyses were restricted to those patients surviving the first year who remained on study beyond the first year, reducing the total sample size to slightly less than one-fourth of the original sample, and eliminating about 60% of the total number of deaths.

The first model, for death from all causes, is shown in Table 21. It is noteworthy that the coefficient for age has been quite similar for the three analyses presented thus far which dealt with patients of both sexes and all ages. The coefficient estimate for the age x group interaction is negative, as in all of the previous analyses, and statistically significant. The coefficients for DBP and cigarettes per day are both negative, as before, with the coefficient estimate for DBP 1.72 standard errors from zero, and the estimate for cigarettes per day only 0.62 standard errors from zero. The reduction in the size of the coefficient for cigarettes per day suggests that not smoking was more of an indicator of poor prognosis for patients dying in the first year than for those dying later. This is somewhat supportive of the theory that those patients who died early in the study were more ill at entry, and less able to obtain or smoke cigarettes.

TABLE 21

Cox Regression Results for the Endpoint
Death from Any Cause for Patients
Who Were Still on Study After One Year

Variables	Coefficient Estimate	Z-ratio
Group Assignment 0=Rx,1=Control	2.8208	2.06
Age	0.0808	8.57
Diastolic Blood Pressure	-0.0121	-1.72
Cigarettes/Day	-0.0081	-0.62
Body Mass Index	-0.0480	-1.95
Diabetes 0=No,1=Yes	-0.1009	-0.40
Sex (0=F,1=M)	0.7442	3.14
Percent of Meals Missed	-0.0674	-1.70
Age x Group	-0.0274	-2.19
BMI x Group	-0.0301	-0.78
Sex x Group	-0.3200	-0.97
% Meals Missed x Group	0.0504	1.14
Baseline Cholesterol	-0.0015	-0.60
Baseline Triglyceride	-0.0018	-0.82
Change in Cholesterol (Baseline-Followup)	0.0074	1.92
Change in Triglyceride (Baseline-Followup)	0.0006	0.23

Maximum log likelihood achieved = -1038.641

Total N = 2072, number of fatal events = 163

The coefficient estimate for the BMI x group interaction has a negative sign, although it is not significant. The coefficient estimate for the main effect of BMI is also negative, just falling short of the -1.96 critical level for significance at the .05 level.

Although the sex x group interaction term is not significant, it suggests that the excess mortality among males in the control group was less than in the diet group. The coefficient estimate for the interaction of compliance and group is also not significant, although it is positive and more than one standard error from zero. The main effect of the percent of meals missed is negative, and has a Z-ratio of -1.70. Although not significant, these results suggest that for patients under study for more than one year, compliance had a negative impact on the diet patients and a much smaller, but still slightly negative impact on the control patients.

Finally, the baseline measures of cholesterol and triglyceride were both negatively associated with mortality, contrary to the results of many of the studies previously described. Both coefficient estimates were less than one standard error from zero however. The average cholesterol at baseline was only 207.2, and only 5% of the sample had pre-diet cholesterol in excess of the 250 mg/dl level considered abnormal in the Merck Manual, so these results are not surprising. The coefficient estimate for change in cholesterol was positive, with a Z-ratio of 1.92, demonstrating that the cholesterol reductions were, in general, counterproductive in reducing mortality from all causes. Change in triglyceride was

estimated to be of little importance in this analysis.

This last analysis suggests, for the first time, that the experimental diet of the MCS may actually have been harmful in some way to patients who were exposed to it for at least one year. It could, of course, also be true that the diet was related to some harmful factor which has not been considered in these models, or which may not have even been measured.

Tables 22 and 23 repeat the analysis of Table 21 for males and females, respectively. There is little new information in these tables, except the observation that the coefficients are somewhat different between the sexes, in general, although the signs are fairly consistent. For males, the coefficient estimate for cigarettes is essentially zero. Also, the conclusions regarding the lipid levels at baseline and the changes in lipids are the same for males as for the total group, except that the coefficient for change in cholesterol is larger for males than for both sexes combined by a factor of two, and the Z-ratio is 2.53. The compliance measure also shows similar results.

For females, shown in Table 23, none of the interactions are significant. The coefficient estimate for BMI is negative and significant. The lipid measures all show an even weaker association with mortality among the females than among the males. Change in triglyceride has a negative sign, suggesting that reductions in triglyceride were beneficial for this subgroup, but the Z-ratio is only -0.61.

TABLE 22

Cox Regression Results for the Endpoint Death from Any Cause for Male Patients Who Were Still on Study After One Year

Variables	Coefficient Estimate	Z-ratio
Group Assignment 0=Rx,1=Control	5.5264	2.90
Age	0.0932	7.19
Diastolic Blood Pressure	-0.0160	-1.62
Cigarettes/Day	0.0011	0.08
Body Mass Index	-0.0154	-0.42
Diabetes 0=No,1=Yes	-0.0380	-0.12
Percent of Meals Missed	-0.0717	-1.44
Age x Group	-0.0439	-2.55
BMI x Group	-0.0990	-1.74
% Meals Missed x Group	0.0339	0.58
Baseline Cholesterol	-0.0044	-1.17
Baseline Triglyceride	-0.0016	-0.50
Change in Cholesterol (Baseline-Followup)	0.0155	2.53
Change in Triglyceride (Baseline-Followup)	0.0033	0.76

Maximum log likelihood achieved = -498.322

Total N = 1100, number of fatal events = 88

TABLE 23

Cox Regression Results for the Endpoint Death from Any Cause for Female Patients Who Were Still on Study After One Year

Variables	Coefficient Estimate	Z-ratio
Group Assignment 0=Rx,1=Control	-0.2787	-0.14
Age	0.0705	4.85
Diastolic Blood Pressure	-0.0071	-0.69
Cigarettes/Day	-0.0632	-1.06
Body Mass Index	-0.0723	-2.12
Diabetes 0=No,1=Yes	-0.2052	-0.51
Percent of Meals Missed	-0.0523	-0.78
Age x Group	-0.0068	-0.34
BMI x Group	0.0283	0.54
% Meals Missed x Group	0.0768	1.05
Baseline Cholesterol	-0.0003	-0.09
Baseline Triglyceride	-0.0012	-0.38
Change in Cholesterol (Baseline-Followup)	0.0025	0.49
Change in Triglyceride (Baseline-Followup)	-0.0022	-0.61

Maximum log likelihood achieved = -422.24

Total N = 972, number of fatal events = 75

Tables 24 and 25 show the Cox regression results for patients younger than 65 years and 65 or older, respectively, for patients alive and still on study after one year. When compared to the total group in Table 21, the younger stratum shows quite similar results, except for some minor differences in the magnitude of the coefficient estimates. The notable exception is that the signs for cholesterol and cholesterol change have reversed, showing cholesterol to be a positive risk factor, and reduction in cholesterol to be beneficial in the younger patients. Both coefficient estimates are less than one standard error from zero, however.

For the older stratum, as for the younger stratum, none of the coefficient estimates for the interactions are significantly different from zero. For those patients 65 years or older, DBP is more of a negative risk factor than for the younger stratum, as is BMI. Compliance with the assigned diet is also apparently more detrimental in the older patients, where the coefficient is more than two times larger in absolute value than for the younger patients. In the older stratum, baseline cholesterol and triglyceride have a rather weak negative association with mortality, and change in cholesterol has a significant positive coefficient, suggesting that reductions in cholesterol were associated with relatively poorer prognosis.

TABLE 24

Cox Regression Results for the Endpoint
Death from Any Cause for Patients Younger Than 65 Years
Who Were Still on Study After One Year

Variables	Coefficient Estimate	Z-ratio
Group Assignment 0=Rx,1=Control	4.7211	1.85
Age	0.0694	2.43
Diastolic Blood Pressure	-0.0067	-0.48
Cigarettes/Day	-0.0111	-0.60
Body Mass Index	-0.0087	-0.17
Diabetes 0=No,1=Yes	0.1037	0.21
Sex (0=F,1=M)	0.6007	1.18
Percent of Meals Missed	-0.0483	-1.01
Age x Group	-0.0457	-1.37
BMI x Group	-0.0926	-1.28
Sex x Group	-0.2364	-0.36
% Meals Missed x Group	0.0429	0.81
Baseline Cholesterol	0.0038	0.76
Baseline Triglyceride	-0.0017	-0.40
Change in Cholesterol (Baseline-Followup)	-0.0058	-0.78
Change in Triglyceride (Baseline-Followup)	0.0049	0.86

Maximum log likelihood achieved = -286.663

Total N = 1537, number of fatal events = 44

TABLE 25

Cox Regression Results for the Endpoint
Death from Any Cause for Patients 65 Years or Older
Who Were Still on Study After One Year

Variables	Coefficient Estimate	Z-ratio
Group Assignment 0=Rx,1=Control	3.1832	1.35
Age	0.0742	4.39
Diastolic Blood Pressure	-0.0118	-1.44
Cigarettes/Day	-0.0006	-0.03
Body Mass Index	-0.0598	-2.06
Diabetes 0=No,1=Yes	-0.1164	-0.39
Sex (0=F,1=M)	0.8195	2.99
Percent of Meals Missed	-0.1034	-1.35
Age x Group	-0.0356	-1.40
BMI x Group	-0.0124	-0.27
Sex x Group	-0.2271	-0.58
% Meals Missed x Group	0.0530	0.59
Baseline Cholesterol	-0.0026	-0.87
Baseline Triglyceride	-0.0021	-0.79
Change in Cholesterol (Baseline-Followup)	0.0108	2.42
Change in Triglyceride (Baseline-Followup)	-0.0006	-0.19

Maximum log likelihood achieved = -650.587

Total N = 535, number of fatal events = 119

XI. COX REGRESSION RESULTS FOR CHD DEATH, RESTRICTED TO PATIENTS SURVIVING THE FIRST YEAR

When the analysis is restricted to patients surviving the first year, only 40 CHD deaths are observed. Therefore, regression coefficients are less likely to have statistical significance, especially when the data are stratified by age and sex. Therefore, in the discussion which will follow, little attention will be focused on the parameter estimates other than those directly related to the diet, that is compliance and the lipid measures. One might have expected that the diet, if truly beneficial in reducing or delaying atherosclerosis, would have shown its most favorable impact on CHD mortality in patients who were under study long enough for their atherosclerosis to be affected. If the diet were not beneficial, or were detrimental in some way, one would also expect this effect to be stronger with longer exposure to the diet.

Table 26 presents the Cox regression results for the CHD death endpoint, for patients surviving the first year. As in most of the preceding analyses, the interaction terms are not significant. While the results are not significant, compliance with either diet is again shown to be negatively associated with mortality, particularly in the diet group. The coefficient for CHD death is almost double that for all causes, but has a larger standard error due to the smaller number of fatal events. The estimate for the main effect is 1.15 standard errors from zero.

Cholesterol and triglyceride have positive coefficients for the

TABLE 26

Cox Regression Results for the Endpoint
CHD Death for Patients
Who Were Still on Study After One Year

Variables	Coefficient Estimate	Z-ratio
Group Assignment 0=Rx,1=Control	4.3441	1.57
Age	0.0643	3.25
Diastolic Blood Pressure	-0.0005	-0.04
Cigarettes/Day	-0.0122	-0.45
Body Mass Index	0.0109	0.21
Diabetes 0=No,1=Yes	0.2343	0.54
Sex (0=F,1=M)	0.8863	1.72
Percent of Meals Missed	-0.1215	-1.15
Age x Group	-0.0258	-1.01
BMI x Group	-0.0819	-1.10
Sex x Group	-0.7920	-1.17
% Meals Missed x Group	0.0700	0.59
Baseline Cholesterol	0.0029	0.60
Baseline Triglyceride	0.0004	0.09
Change in Cholesterol (Baseline-Followup)	0.0034	0.46
Change in Triglyceride (Baseline-Followup)	0.0009	0.17

Maximum log likelihood achieved = -255.852

Total N = 2072, number of fatal events = 40

CHD death endpoint, but both are of low precision, neither Z-ratio exceeding 1. Change in cholesterol and triglyceride both have positive coefficients that are not statistically significant. Thus even for the CHD death endpoint, there is evidence that being on the diet and thereby reducing serum cholesterol was not beneficial, and may have been counterproductive.

Table 27 gives the Cox regression results for the CHD death endpoint for male patients who were on study for at least one year. Although neither the interaction nor the main effect are significant, the results again suggest that compliance was detrimental for males, although more so for patients in the control group in this case. Cholesterol and triglyceride again have the "wrong" sign, that is they are negatively associated with mortality. For males, the coefficient for change in cholesterol is positive and highly significant.

For females, as shown in Table 28, cholesterol and triglyceride both show a positive association with CHD mortality, and change in cholesterol has a negative sign, indicating that female patients with relatively larger drops in cholesterol enjoyed a more favorable mortality experience from CHD. Aside from age, the only coefficient estimate that exceeds its standard error is change in cholesterol, with a Z-ratio of -1.08.

In the thirteen patients under the age of 65 who survived the first year but who later died of CHD, shown in Table 29, baseline cholesterol has a relatively strong positive association with CHD

TABLE 27

Cox Regression Results for the Endpoint
CHD Death for Male Patients
Who Were Still on Study After One Year

Variables	Coefficient Estimate	Z-ratio
Group Assignment<br>0=Rx,1=Control	8.3208	2.19
Age	0.0689	2.47
Diastolic Blood Pressure	0.0040	0.22
Cigarettes/Day	-0.0018	-0.06
Body Mass Index	0.0127	0.17
Diabetes<br>0=No,1=Yes	0.1653	0.25
Percent of Meals Missed	-0.3467	-1.37
Age x Group	-0.0431	-1.14
BMI x Group	-0.1876	-1.60
% Meals Missed x Group	-0.2959	-0.72
Baseline Cholesterol	-0.0016	-0.21
Baseline Triglyceride	-0.0014	-0.20
Change in Cholesterol<br>(Baseline-Followup)	0.0305	2.41
Change in Triglyceride<br>(Baseline-Followup)	-0.0038	-0.47

Maximum log likelihood achieved = -103.747

Total N = 1100, number of fatal events = 20

TABLE 28

Cox Regression Results for the Endpoint
CHD Death for Female Patients
Who Were Still on Study After One Year

Variables	Coefficient Estimate	Z-ratio
Group Assignment 0=Rx,1=Control	1.7739	0.46
Age	0.0666	2.15
Diastolic Blood Pressure	-0.0071	-0.36
Cigarettes/Day	-0.0115	-0.21
Body Mass Index	0.0132	0.19
Diabetes 0=No,1=Yes	0.2172	0.36
Percent of Meals Missed	-0.0173	-0.19
Age x Group	-0.0149	-0.38
BMI x Group	-0.0216	-0.24
% Meals Missed x Group	0.0358	0.34
Baseline Cholesterol	0.0043	0.64
Baseline Triglyceride	0.0026	0.53
Change in Cholesterol (Baseline-Followup)	-0.0094	-1.08
Change in Triglyceride (Baseline-Followup)	0.0013	0.19

Maximum log likelihood achieved = -114.993

Total N = 972, number of fatal events = 20

TABLE 29

Cox Regression Results for the Endpoint
CHD Death for Patients Younger Than 65 Years
Who Were Still on Study After One Year

Variables	Coefficient Estimate	Z-ratio
Group Assignment 0=Rx,1=Control	7.6469	1.38
Age	0.0835	1.20
Diastolic Blood Pressure	-0.0081	-0.31
Cigarettes/Day	-0.0231	-0.60
Body Mass Index	0.0360	0.38
Diabetes 0=No,1=Yes	1.2147	1.84
Sex (0=F,1=M)	0.5314	0.55
Percent of Meals Missed	-0.0231	-0.26
Age x Group	-0.0889	-1.16
BMI x Group	-0.0956	-0.76
Sex x Group	-0.3400	-0.29
% Meals Missed x Group	0.0043	0.04
Baseline Cholesterol	0.0140	1.57
Baseline Triglyceride	0.0000	0.00
Change in Cholesterol (Baseline-Followup)	-0.0066	-0.48
Change in Triglyceride (Baseline-Followup)	0.0016	0.19

Maximum log likelihood achieved = -78.199

Total N = 1537, number of fatal events = 13

mortality, with a Z-ratio of 1.57. Change in cholesterol has a negative sign, indicating that cholesterol lowering was beneficial, but the coefficient is small relative to its standard error. This, and the previous results, suggest that the beneficial effects of the diet, if they existed, were confined to younger patients.

Table 30 further emphasizes this last point, but here the sample size and number of events is too small for the results to be very reliable. For the older stratum of patients, the estimated effect of compliance is rather similar for the two treatment groups. The main effect for the percent of meals missed has a very large negative coefficient, though not a significant one. Cholesterol and change in cholesterol have the same reversed signs that were shown for all patients combined and for male patients.

Many of the previous tables have suggested that the experimental diet of the MCS, and reductions in cholesterol that resulted from the diet, were counterproductive. Compliance with either diet was generally associated with increased mortality, and this negative effect was usually greater among the diet patients. Also, cholesterol reductions were generally associated with increased mortality, especially among males and older patients. The notion of compliance being a measure of mobility was introduced earlier, but the differential effect in the two treatment groups would seem to imply that something in the experimental diet was detrimental. A more sensitive means for studying this possibility is to confine the analysis to patients in the diet group who survived the first year.

TABLE 30

Cox Regression Results for the Endpoint CHD Death for Patients 65 Years or Older Who Were Still on Study After One Year

Variables	Coefficient Estimate	Z-ratio
Group Assignment 0=Rx,1=Control	4.0661	0.83
Age	0.0345	0.89
Diastolic Blood Pressure	0.0044	0.28
Cigarettes/Day	0.0033	0.08
Body Mass Index	0.0052	0.08
Diabetes 0=No,1=Yes	-0.2626	-0.40
Sex (0=F,1=M)	1.0076	1.62
Percent of Meals Missed	-0.4998	-1.30
Age x Group	-0.0191	-0.35
BMI x Group	-0.0874	-0.94
Sex x Group	-0.6446	-0.76
% Meals Missed x Group	0.0276	0.05
Baseline Cholesterol	-0.0011	-0.18
Baseline Triglyceride	0.0006	0.11
Change in Cholesterol (Baseline-Followup)	0.0061	0.68
Change in Triglyceride (Baseline-Followup)	-0.0005	-0.07

Maximum log likelihood achieved = -145.825

Total N = 535, number of fatal events = 27

This analysis is presented in Table 31, and is comparable to adding interaction terms for the lipid measures to the basic models used previously. For the diet patients, the coefficient for percent of meals missed is slightly more negative than in Table 21, and the baseline lipid levels demonstrate a negative effect similar to that for both treatment groups combined. The coefficient estimate for change in cholesterol also shows the same "backward" relationship, but for triglyceride change, the association is in the opposite direction. Table 32 shows results analogous to those of the previous table for the CHD death endpoint. The results are essentially the same, showing no beneficial effect, and hints of a harmful effect of the experimental diet.

TABLE 31

Coefficient Estimates for the Cox Regression Model for the Endpoint Death from Any Cause, for Patients on the Experimental Diet Who Were Still on Study After One Year

Variables	Coefficient Estimate	Z-ratio
Age	0.0856	8.71
Diastolic Blood Pressure	0.0010	0.10
Cigarettes/Day	0.0105	0.71
Body Mass Index	-0.0503	-2.00
Diabetes 0=N,1=Y	-0.1534	-0.44
Percent of Meals Missed	-0.0752	-1.87
Sex 0=Female,1=Male	0.6616	2.71
Baseline Cholesterol	-0.0014	-0.35
Baseline Triglyceride	-0.0030	-0.98
Change in Cholesterol (Baseline-Followup)	0.0087	1.51
Change in Triglyceride (Baseline-Followup)	-0.0038	-1.24

Maximum log likelihood achieved = -466.866

Total N = 1039, number of deaths = 85

TABLE 32

Coefficient Estimates for the Cox Regression Model for the Endpoint Death from CHD Causes, for Patients on the Experimental Diet Who Were Still on Study After One Year

Variables	Coefficient Estimate	Z-ratio
Age	0.0716	3.46
Diastolic Blood Pressure	0.0142	0.72
Cigarettes/Day	0.0065	0.19
Body Mass Index	0.0147	0.29
Diabetes 0=N,1=Y	0.2811	0.42
Percent of Meals Missed	-0.1306	-1.20
Sex 0=Female,1=Male	0.6657	1.25
Baseline Cholesterol	-0.0023	-0.26
Baseline Triglyceride	-0.0057	-0.81
Change in Cholesterol (Baseline-Followup)	0.0093	0.73
Change in Triglyceride (Baseline-Followup)	-0.0059	-0.96

Maximum log likelihood achieved = -98.523

Total N = 1039, number of deaths = 18

XII. SUMMARY AND CONCLUSIONS

When the Cox regression coefficients for all the models presented thus far are compared, some patterns emerge. While there is a problem with low precision for many of the estimates, the consistency of some of the trends is noteworthy. In spite of the lack of statistical significance, the regression results should not be rejected out of hand. The estimates are the "most likely" values for the coefficients, based on the data. If an estimate is only one standard error from zero in the negative direction, for example, the probability that one would observe such a result if the true value of the coefficient were zero is less than .32, using a two-tailed test. Also, with such an observation, one feels more confident that the true value for the coefficient is not likely to be positive. Most of the discussion so far has focused on the direction, rather than the strength of the associations. Results for each of the important independent variables will be summarized below.

In all regressions the age x group interaction coefficients were negative, which seems to imply that the effect of aging was more severe in the diet group. The reason for this finding is not clear. Age always showed a positive coefficient estimate, as one would expect.

DBP was negatively associated with mortality from all causes in all the regressions, but was positively associated with CHD mortality for most of the regressions for that endpoint. The effect of DBP was small in all of the regressions.

In all but two of the regressions, cigarette smoking showed a negative association with mortality. For the entire cohort of patients the coefficient estimate was significant, even when stratified by age and sex. For the CHD death endpoint for the entire cohort, the coefficients were also all negative, but smaller in absolute value relative to the corresponding result for all causes of mortality, and none of these coefficients were significant. These results are clearly at odds with many previous studies. Table 3 indicated that about 43% of the patients were smokers. Table 33 shows the cigarette smoking characteristics of MCS patients by age and sex. In general, the MCS men are heavier smokers than men in a National Clearinghouse for Smoking and Health survey conducted in 1970. Table 34 compares the smoking characteristics of the MCS patients who died while on study to the patients who survived. The patients who died were much less likely to have been smokers at entry, even when the data are stratified by age. For patients who did smoke, the number of cigarettes consumed per day was similar for deaths and survivors. This suggests that the difference in smoking habits might have been related to health, that is, the less healthy patients with higher mortality were unable to obtain or smoke cigarettes. The data contain no overall measure of health, mental or physical, so more detailed exploration of this idea is not possible.

TABLE 33

Cigarette Smoking Characteristics of the MCS Population by Age and Sex

Age	Cigs/Day	Percent Smokers		Cigs/Day for Smokers
LT 35				
Males	14.7	60.6	(48.0)*	24.2
Females	12.3	52.3		23.6
35 - 44				
Males	15.4	59.9	(48.6)	25.7
Females	11.9	51.3		23.1
45 - 54				
Males	11.2	52.2	(43.2)	21.5
Females	9.2	44.2		20.9
55 - 64				
Males	8.5	46.9	(37.5)	18.1
Females	4.4	25.3		17.5
GE 65				
Males	3.6	23.2	(23.0)	15.4
Females	1.0	6.2		15.3

* Parenthetical numbers are the percent of smokers among a sample of males surveyed by the National Clearinghouse for Smoking and Health in 1970.

TABLE 34

Smoking Characteristics of the MCS Population
by Age and Survival Status

	Death from Any Cause		Survivors	
Age (yrs.)	% Smokers	Cigs/Day for Smokers	% Smokers	Cigs/Day for Smokers
LT 35	9.1	20.0	57.0	23.9
35 - 44	0.0	0.0	55.3	24.3
45 - 54	17.9	24.0	48.0	21.1
55 - 64	20.6	16.7	36.6	17.9
GE 65	9.0	12.5	15.6	15.7

Another MCS result which was somewhat of a surprise was the consistent negative regression coefficient for BMI for death from all causes. The coefficient estimates were significant for the entire cohort and for males and patients older than 65 years. For the CHD death endpoint, the coefficient estimates were most often positive, but never statistically significant. The BMI x group interaction was always negative, and the combined coefficient estimates for the main effect and interaction suggest that the negative effect of BMI was stronger in the control group. Table 3 showed the mean body mass index to be 24.5 kg/m^2. About one-fourth of the patients had BMI less than or equal to 20, which would correspond, for example, to a person who was six feet tall weighing less than 147 pounds. As alluded to earlier, the negative effect of BMI could reflect the debilitated state of some of the patients, particularly the older male patients, rendering BMI something of an inverse measure of fitness. The average BMI for the entire cohort suggests that the population was, at least on the average, not overweight.

In all of the regressions, males experienced heavier mortality than females. There was no consistent pattern to indicate that the excess mortality in males was more pronounced in one treatment group than in the other.

Compliance with the assigned diet was associated with heavier mortality in the control group than in the diet group in all the regressions, except those restricted to females. Also, the

coefficient for CHD death was always smaller than that for death from all causes. This indicates that compliance with the control diet had its most unfavorable impact on CHD mortality. This could suggest that the control diet was more atherogenic than whatever the control patients consumed outside the dining facility. This is sheer speculation, however, since data to support this hypothesis were not collected.

For the diet patients, compliance was also associated with heavier mortality, with a few exceptions. The exceptions were the entire cohort for death from all causes, females for both endpoints, and patients 65 or older for the combined endpoint. For all patients, for males, and for older patients, the coefficient for death from CHD is more negative than for death from all causes, just as observed for the control group. This was true even when the analysis was restricted to patients still in the hospital after one year.

When the effect of compliance between the two groups is compared in each of the regressions, no clear pattern emerges. Sometimes the coefficient estimate for controls exceeds that for diet patients, and sometimes the opposite is true. It is interesting to note that the coefficient estimate for each regression that was restricted to patients still in the hospital after one year is more negative than the coefficient estimate for the corresponding regression without the restriction. This might be interpreted as additional evidence of a negative dose effect of the diet. Again, it is impossible to

attach much significance to these results, since the precision is relatively low, however two possibilities must be considered. The first is that compliance to the diet was directly detrimental because of toxicity. The second possibility is that compliance, like cigarette smoking, is a measure of relative fitness and ability to get out of the institution. It is known, for example, that patients who were unable to go to the dining room had trays of food brought to them from the kitchen, and thus their compliance was good, while their health was, in general, poor. Compliance was related to age, the average age of patients attending 90% or more of the meals being 56.3 years, while the average age of patients attending less than 90% of the meals was 40.8 years.

Baseline cholesterol was not shown to be associated in any important way with mortality in the MCS. The coefficient estimate for this variable was usually negative, although it was positive for CHD death, and for patients less than 65 years old for both endpoints. Reductions in cholesterol were only estimated to be beneficial in the cases of females for the CHD death endpoint and for patients under 65 for both endpoints. Triglyceride and change in triglyceride showed an even weaker association than cholesterol and change in cholesterol.

There are a number of tentative conclusions that can be drawn from the MCS data. The first of these is not really tentative at all, and is that compliance with a diet like that of the MCS can greatly reduce serum cholesterol. However, the data do not suggest

that this reduction in cholesterol had a beneficial effect on mortality, and in fact there is every indication that it increased mortality slightly, particularly among older patients. Other investigators have done preliminary analyses which indicated that the incidence of cancer deaths in the MCS is not responsible for the excess mortality in the diet group. It will be recalled that the results of some earlier studies that employed diets high in polyunsaturated fats suggested increased incidence of cancer death.

No global conclusions should be derived from the estimated coefficients for any of the factors, since the MCS population is certainly atypical of a general population with regard to mental health, whatever effect that might have had on the outcome, but also with regard to physical health and other characteristics, such as smoking. Indices of mental and overall physical health that could have been used in the analysis would have been useful, as might have been additional exclusions at the time of randomization. Unfortunately, most of the information provided by this experiment was derived from older patients who tended to have longer lengths of stay, and also higher mortality. It is unlikely that one would consider actually promoting a diet of this type in the older age groups of a free-living population, and as alluded to earlier, compliance in any free-living population would be a special problem.

In spite of all the previous caveats, another possibility must be considered. That is the possibility that serum cholesterol can be modified by dietary means, but the incidence of death from CHD and

other causes cannot. To date, the strongest evidence that CHD incidence can be reduced by dietary intervention has been provided by the Finnish Mental Hospital study, and their results for death from all causes were not significant. If only the manner and not the rate of death can be modified, then perhaps such dietary interventions and changes of lifestyle are not worthwhile. Several other studies have shown inconclusive results, and the MCS may be additional evidence to refute the diet hypothesis.

Finally, it is possible that the hypothesis has not had an adequate test. If diet could reduce CHD incidence it would probably be through one of two mechanisms. Either atherosclerosis would be halted or retarded, or else it might be reversed. There exists no evidence that reversal, if it occurs, would show beneficial effects in the three to six years that diet studies typically last. Perhaps a study in a general population over a long period of time is needed to adequately test the diet hypothesis. In any case, the results of the Minnesota Coronary Survey must be considered inconclusive.

Appendix A

Analysis of MCS Censoring Data Using Lifetable Methods

As alluded to in the main body of this thesis, an understanding of the censoring mechanism is useful in interpreting the mortality data, especially where censoring is severe. In complete analogue to the mortality analysis, censoring here will be considered to be the event of primary interest, and death to be withdrawal prior to the occurrence of the event. The hazard function, defined previously as the force of mortality, can be considered to be the force of censoring in this application. The Kaplan-Meier life table is applicable to the censoring data, the values displayed and plotted representing the cumulative proportion of censored individuals.

Censoring occurred in two ways in the MCS. The first type of censoring occurred when a patient left the hospital prior to the termination of the study. The second type of censoring occurred when a patient was randomized after the earliest possible date of randomization, and so had less than the maximum possible length of follow-up. Many patients were in fact subject to both methods of censoring. In the Kaplan-Meier analysis, it is only of interest to know whether censoring was more severe in one randomization group than in the other, without regard to how the censoring occurred. It is really the first method of censoring that is critical, since the randomization should have provided approximately equal rates of accrual, and so any differences in censoring are likely to be due to withdrawal prior to the official termination date. The Kaplan-Meier

life table for censoring is presented in Table A-1, and plotted in Figure A-1.

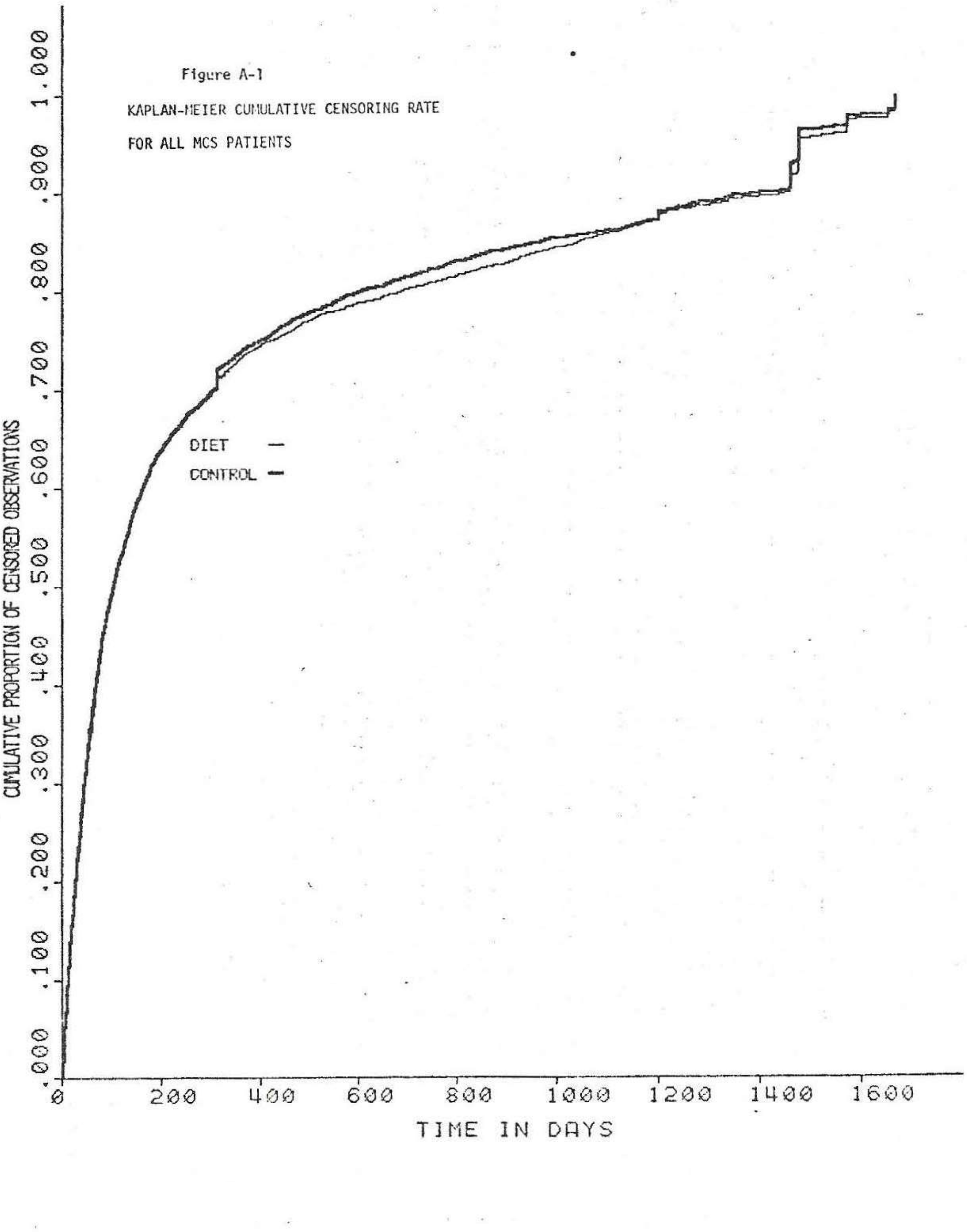

Figure A-1

KAPLAN-MEIER CUMULATIVE CENSORING RATE

FOR ALL MCS PATIENTS

TABLE A-1

Cumulative Rate of Censoring as Estimated by Kaplan-Meier for All MCS Patients

Interval $t_i - t_{i+1}$ (yrs)	Control: No. at Risk at t_i	Control: $\hat{F}(t_i)$	Diet: No. at Risk at t_i	Diet: $\hat{F}(t_i)$	Z*
0 - 1	4738	0	4685	0	-
1 - 2	1153	.7413	1165	.7350	0.68
2 - 3	770	.8206	802	.8074	1.61
3 - 4	564	.8625	558	.8597	0.38
4 - 5	275	.9299	310	.9184	1.98

* Ratio of difference in cumulative censoring (control-diet) to the standard error of the difference

Figure A-1 shows that nearly half of the observations had been censored by 100 days. Also, censoring was nearly equal in the two groups up to about one year. At that point, there was a sharp jump in the censoring rate in both groups, with the jump more pronounced in the control group. Several more of these jumps occurred at approximately 1200, 1460, 1480 and 1580 days. These jumps are the result of the simultaneous censoring of a number of individuals as a result of their entering the study on the first possible day for a given hospital, and remaining on study through the last day. At the beginning of the fourth year the censoring was significantly greater in the control group, although the sample at risk of being censored at that point was very small. There is no evidence that censoring was more severe for one group than for the other in the first three

years of the study.

Appendix B

Under the Peto and Breslow approximation to the likelihood of the basic model proposed by Cox, the log likelihood can be expressed as

$$L(B) = \sum_{i=1}^{n} s_{(i)} B - \sum_{i=1}^{n} m_i \log \sum_{\ell \varepsilon R} \exp(z_{(\ell)} B)$$

where $s_{(i)}$ is the sum of the covariate values for all subjects in the risk set at $t_{(i)}$, and $m_{(i)}$ is the number of subjects who failed at $t_{(i)}$. As mentioned earlier, the first and second partial derivatives are obtained and set equal to zero. The resulting normal equations are solved to obtain the estimates for B. The first and second derivatives of L(B) are as follows:

$$\frac{\partial L(B)}{B_k} = \Sigma\, s_{k(i)} - \sum_i m_i \frac{\sum_\ell z_{k(\ell)} \exp(z_{(\ell)} B)}{\sum_{\ell \varepsilon R} \exp(z_{(\ell)} B)}$$

$$\frac{\partial^2 L(B)}{\partial B_j\, \partial B_k} = - \Sigma\, m_i \left[\frac{\Sigma\, z_{k(\ell)} z_{j(\ell)} \exp(z_{(\ell)} B)}{\Sigma \exp(z_{(\ell)} B)} \right.$$

$$\left. - \frac{\Sigma\, z_{k(\ell)} \exp(z_{(\ell)} B)\ \Sigma\, z_{j(\ell)} \exp(z_{(\ell)} B)}{\Sigma \exp(z_{(\ell)} B)\ \Sigma \exp(z_{(\ell)} B)} \right],$$

where the summation is performed at each point in time at which at least one failure occurs.

Appendix C

Programming Considerations for Cox Regression and Other Maximum Likelihood Problems

A major problem in using the Cox regression method on a large data set, and in fact a problem in most maximum likelihood estimation procedures is the storing of the covariate values need for the computations. Typically, the covariates are need at least once for each iteration, and in the case of Cox regression they are needed several times in each iteration. The two usual alternatives, internal storage and external, or scratch storage, both have practical limitations. Internal storage requires large amounts of central memory. The MCS data consisted of over 9000 cases, and some problems considered more than 10 covariates for each case. Internal storage then would require a minimum of 90,000 words of central memory, with nearly that amount again needed for storing the failure time and the survival status, as well as the results of intermediate calculations and the program itself. Many computers do not have central memories that can accomodate such programs, and others could only be used at special non-peak usage times, and at oftentimes great cost.

External storage, the writing of the covariates for each case to a scratch file, and the reading of the file as many times as needed for each iteration is also feasible, but the enormous number of I/O operations would be extremely costly in terms of computer time. Also, non-sequential access to cases is very complicated, usually

requiring an indexed file.

To circumvent these problems in the present case of the MCS data, two subroutines were written to pack numerically the survival time, survival status, and the covariates into as few locations as possible for internal storage. All covariates were considered to be integer values, and the length of each variable in digits was provided to the subroutines via a DATA statement. When the data had been read from the masterfile, they were passed to the first subroutine, called PACK. The Honeywell 66/10 computer allows storage of integers up to 10 digits in length. The first variable, of length l_1 , was then packed into the leftmost l_1 digits of the first integer word by replacing that integer word by the value of the variable multiplied by the appropriate power of ten. This was continued for the next variable, the value being multiplied by some smaller power of ten and added to the previous value of the first storage word. When all ten digits of the word had been used, a second word was used, and so on, until all the covariates had been packed. Provision was made for covariate values to be spread over two words if needed. The packing was done once for each subject and required little computer time, since only arithmetic operations were used. A problem which would have required 100,000 storage locations could now be reduced to perhaps 50,000 locations, still a large amount of storage, but certainly more manageable.

Unpacking of the data was accomplished through the subroutine UNPACK, which essentially reversed the operation of PACK, dividing

by appropriate powers of ten, truncating, and subtracting. Again, the numerical operations made access quite efficient, and the internal storage made case access on a non-sequential basis possible.

Problems which were anticipated were the presence of negative values, and the possibility of non-integral values. Negative values were handled by assigning a binary flag to whether a particular variable was negative, and packing the flag and the absolute value of the variable. This kind of procedure increases the chance of programmer error, so printing of some of the values for a number of cases before and after packing and unpacking is suggested. Non-integral values may also be packed, provided a scale factor is provided and only a fixed number of digits to the right of the decimal point are retained. The scale factor is applied before packing and after unpacking.

While this procedure can be prone to error and has other limitations, it makes solution of large maximum likelihood problems possible on many computers where it might otherwise be impossible. Listings of the two subroutines, and an example follow.

EXAMPLE

	Value for this case	Max. length
Variable No. 1	17	3
2	1	1
3	2692	4
4	-97	3
5	32	3

Variable 4 is replaced by its absolute value, and Variable 6 is added to reflect the negative sign. PACK is called, and the data are stored, not in 5 locations as would normally be required, but in 2 locations as follows.

Location 1

[ ] [1] [7] [1] [2] [6] [9] [2] [0] [9]

Location 2

[7] [0] [3] [2] [1] [ ] [ ] [ ] [ ] [ ]

SUBROUTINE LISTINGS

Variables are defined as follows:

NVAR is the number of distinct data items to be packed or unpacked, including flags for negative values, survival times, and survival indicator flag.

DATA is an array of data items to be packed or unpacked. Note that DATA has NVAR elements.

IOBS is the number of the case to be packed or unpacked.

STORAGE(K,IOBS) is the address for the kth word of packed data for the case indexd by IOBS. STORAGE is passed through COMMON.

LNG is an array of lengths for each data item. LNG must be of dimension NVAR.

```
      SUBROUTINE PACKI(NVAR,NWORDS,LNG)
      INTEGER TLNG,SUM
      DIMENSION LNG(1)
      DO 10  J=1,NVAR
10    TLNG=TLNG+LNG(J)
      JWORDS=(TLNG+8)/9
      IF(NWORDS.LT.JWORDS)  STOP 0077
      PRINT 800, JWORDS
800   FORMAT(1X,"WORDS NEEDED FOR COMPLETE STORAGE = NCASES*"I2)
      NWORDS = JWORDS
      RETURN
      END
```

```
      SUBROUTINE PACK(NVAR,DATA,STORAGE,LNG,IOBS)
      INTEGER STORAGE,SUM
      DIMENSION DATA(1),STORAGE(1,1),LNG(1)
      J=1
      STORAGE(1,IOBS)=0
      SUM=0
      DO 50  I=1,NVAR
      SUM=SUM+LNG(I)
      IF(SUM.GT.9)  GO TO 30
20    STORAGE(J,IOBS)=STORAGE(J,IOBS)+DATA(I)*10**(9-SUM)
      IF(SUM.EQ.9)  GO TO 40
      GO TO 50
30    ITEMP=(DATA(I)+.001)/10**(SUM-9)
      JTEMP=DATA(I)-ITEMP*10**(SUM-9)
      STORAGE(J,IOBS)=STORAGE(J,IOBS)+ITEMP
      DATA(I)=JTEMP
      SUM=SUM-9
      J=J+1
      STORAGE(J,IOBS)=0
      GO TO 20
40    J=J+1
      STORAGE(J,IOBS)=0
      SUM=0
50    CONTINUE
      RETURN
      END
```

```
      SUBROUTINE UNPACK(NVAR,DATA,STORAGE,LNG,IOBS)
      INTEGER STORAGE,REST
      DIMENSION DATA(1),STORAGE(1,1),LNG(1)
      J=1
      SUM=0
      REST=0
      DO 50  I=1,NVAR
      IBFLG=0
      SUM=SUM+LNG(I)
      NEXT=0
      IF(I.LT.NVAR)  NEXT=LNG(I+1)
      IF(SUM+NEXT.GT.9.)  NEXT=9-SUM
      LNGT=LNG(I)
      IF(SUM.GT.9)  GO TO 100
   20 IDATA=(STORAGE(J,IOBS)+.9)/10**(9-SUM)
      DATA(I)=IDATA-REST
      REST=(STORAGE(J,IOBS)+.9)/10**(9-SUM)
      REST=REST*10**NEXT
      IF(IBFLG.EQ.1)  GO TO 150
      IF(IBFLG.EQ.2)  GO TO 200
      GO TO 50
C *******NEXT VARIABLE CROSSES WORD BOUNDARY**************
  100 LNGREM=SUM-9
      LNGT=LNGT-LNGREM
      IBFLG=1
      SUM=9
      NEXT=0
      GO TO 20
  150 J=J+1
      LEFT=DATA(I)
      SUM=LNGREM
      REST=0
      LNGT=LNGREM
      NEXT=LNG(I+1)
      IBFLG=2
      GO TO 20
  200 DATA(I)=DATA(I)+LEFT*10**LNGREM
   50 CONTINUE
      RETURN
      END
```

BIBLIOGRAPHY

Anitschkow, N.(1933). Experimental Arteriosclerosis in Animals. In Arteriosclerosis, edited by Cowdry, E. V., Macmillan, New York, 271.

Brown, J., Bourke, G. J., Gearty, G. F., Finnegan, A., Hill, M., Hefferman-Fox, F. C., Fitzgerald, D. E., Kennedy, J., Childers, R. W., Jessop, W. J. E., Trulson, M. F., Latham, M. C., Cronin, S., McCann, M. B., Clancy, R. E., Gore, I., Stoudt, H. W., Hegsted, D. M. and Stare, F. J., (1970). Nutritional and Epidemiological Factors Related to Heart Disease. In World Review of Nutrition and Dietetics, Vol. 12, edited by G. Bourne, p.l. Karger, Basel, New York.

Cornfield, J., Gordon, T. and Smith, W. W.(1961). Quantal Response Curves for Experimentally Uncontrolled Variables. Bull. Inst. Int. Statist. 38, 97-115.

Cox, D. R.(1972). Regression Models and LIfe Tables. J. R. Stat. Soc. B. 34, 187-220.

Dayton, S., Pearce, M. L., Hashimoto, S., Dixon, W., and Tomiyasu, U.(1969). A Controlled Clinical Trial of a Diet High in Unsaturated Fat in Preventing Complications of Atherosclerosis. Circulation 40, Supplement No. 2.

Feigl, P. and Zelen, M.(1965). Estimation of Exponential Survival Probabilities with Concommitant Information. Biometrics 21, 826-837.

Hardinge, M. G. and Stare, F. J.(1954). Nutritional Studies of Vegetarians. 2. Dietary and Serum Levels of Cholesterol in Man. Am. J. Clin. Nutr. 17, 281-295.

Kagan, A., Harris, B. R., Winkelstein, W., Jr., Johnson, K. G., Kato, H., Syme, S. L., Rhoads, G. G., Gay, M. L., Nichaman, M. Z., Hamilton, H. B., and Tillotson, J.(1974). Epidemiologic Studies of Coronary Heart Disease and Stroke in Japanese Men Living in Japan, Hawaii and California: Demographic, Physical, Dietary and Biochemical Characteristics. J. Chron. Dis. 27, 345-364.

Kaplan, E. L., Meier, P.(1958). Nonparametric Estimation from Incomplete Observations. JASA 53, 457-480.

Katz, L. N. and Stamler, J.(1953). Experimental Atherosclerosis. Charles C. Thomas, Springfield, Illinois.

Keys, A.(1953). Atherosclerosis: A Problem in Newer Public Health. J. Mt. Sinai Hosp. 20, 118-139.

Kimura, N.(1956). Analysis of 10,000 Postmortem Examinations in Japan. In World Trends in Cardiology: I. Cardiovascular Epidemiology, Edited by A. Keys and P. D. White,p.159. Hoeber-Harper, New York.

Malmros, H.(1950). The Relation of Nutrition to Health - A Statistical Study of the Effect of War-Time on Arteriosclerosis, Cardiosclerosis, Tuberculosis, and Diabetes. Acta Med. Scand., Suppl. 246, 137-153.

Mantel, N. and Myers, M.(1971). Problems of Convergence of Maximum Likelihood Iterative Procedures in Multiparameter Situations. JASA 66, 484-491.

McGee, D. and Gordon, T.(1976). The Framingham Study - An Epidemiological Investigation of Cardiovascular Disease, Section 31. The Results of the Framingham Study Applied to Four Other U. S. Based Epidemiologic Studies of Cardiovascular Disease, U. S. Department of Health, Education and Welfare, DHEW Publication No. (NIH) 76-1083, Washington, D. C.

The Merck Manual of Diagnosis and Therapy(1972). Edited by D. Holvey, Merck Sharp and Dohme Research Laboratories, p.1792.

Moriyama, I. M., Krueger, D. E., and Stamler, J.(1971). Cardiovascular Diseases in the United States. Harvard University Press, Cambridge, Massachusetts.

National Clearinghouse for Smoking and Health, U.S. Department of Health, Education and Welfare(1973). Adult Use of Tobacco.

National Diet-Heart Study Research Group(1968). The National Diet-Heart Study Final Report. Circulation 37, Supplement No. 1.

National Heart, Blood Vessel, Lung, and Blood Program, Second Annual Report of the Director of the National Heart and Lung Institute, U.S. Department of Health, Education and Welfare(1975).

Pearce, M. L., and Dayton, S.(1970). Incidence of Cancer in Men on a Diet High in Unsaturated Fat. Abstracts of the 43rd Scientific Sessions of the American Heart Association, Circulation 41 and 42, Supplement No. 3, p.52.

Phillips, R., Lemon, F.,Beeson, W., and Kuzma, J.(1978). Coronary Heart Disease Mortality among Seventh-Day Adventists with Differing Dietary Habits. Am. J. Clin. Nutr. 31, Supplement, S191.

Rosenthal, S. R.(1934). Studies in Atherosclerosis: Chemical, Experimental, and Morphologic. Arch. Path. 18, 473-506, 660-698 and 827-842.

Turpeinen, O.(1979). Effect of Cholesterol-Lowering Diet on Mortality from Coronary Heart Disease and Other Causes. Circulation 59, 1-7.

Vital Statistics of the United States, 1975, Volume II - Mortality, Part A, U.S. Department of Health, Education and Welfare, Table 1-7.

Walker, S. H. and Duncan, D. B.(1967). Estimation of the Probability of an Event as a Function of Several Independent Variables. Biometrika 54, 167-178.

Worth, R. M., Kato, H., Rhoads, G. G., Kagan, A. and Syme, S. L.(1975). Epidemiologic Studies of Coronary Heart Disease and Stroke in Japanese Men Living in Japan, Hawaii and California: Mortality. Am. J. Epid. 102, 481-490.

Yerushalmy, J. and Hilleboe, H. E.(1957). Fat in the Diet and Mortality from Heart Disease - A Methodological Note. New York State J. Med. 57, 2343-2354.

Zippin, C. and Armitage, P.(1966). Use of Concommitant Variables and Incomplete Survival Information in the Estimation of an Exponential Survival Parameter. Biometrics 22, 665-672.

MnU-M
81-18
APR 9 '82
